# Host immune constraints on malaria transmission: insights from population biology of within-host parasites

**DOI:** 10.1186/1475-2875-12-206

**Published:** 2013-06-15

**Authors:** Philip G McQueen, Kim C Williamson, F Ellis McKenzie

**Affiliations:** 1 Mathematical and Statistical Computing Laboratory, Division of Computational Bioscience, Center for Information Technology, National Institutes of Health, Bethesda, Maryland, USA; 2 Department of Biology, Chicago, Loyola University of Chicago, Chicago, Illinois, USA; 3 Laboratory of Malaria and Vector Research, National Institute of Allergy and Infectious Diseases, National Institutes of Health, Bethesda, Maryland, USA; 4 Fogarty International Center, National Institutes of Health, Bethesda, Maryland, USA

## Abstract

**Background:**

*Plasmodium* infections trigger complex immune reactions from their hosts against several life stages of the parasite, including gametocytes. These immune responses are highly variable, depending on age, genetics, and exposure history of the host as well as species and strain of parasite. Although the effects of host antibodies that act against gamete stages in the mosquito (due to uptake in the blood meal) are well documented, the effects of host immunity upon within-host gametocytes are not as well understood. This report consists of a theoretical population biology-based analysis to determine constraints that host immunity impose upon gametocyte population growth. The details of the mathematical models used for the analysis were guided by published reports of clinical and animal studies, incorporated plausible modalities of immune reactions to parasites, and were tailored to the life cycl es of the two most widespread human malaria pathogens, *Plasmodium falciparum* and *Plasmodium vivax*.

**Results:**

For the same ability to bind and clear a target, the model simulations suggest that an antibody attacking immature gametocytes would tend to lower the overall density of transmissible mature gametocytes more than an antibody attacking the mature forms directly. Transmission of *P. falciparum* would be especially vulnerable to complete blocking by antibodies to its immature forms since its gametocytes take much longer to reach maturity than those of *P. vivax*. On the other hand, antibodies attacking the mature gametocytes directly would reduce the time the mature forms can linger in the host. Simulation results also suggest that varying the standard deviation in the time necessary for individual asexual parasites to develop and produce schizonts can affect the efficiency of production of transmissible gametocytes.

**Conclusions:**

If mature gametocyte density determines the probability of transmission, both *Plasmodium* species, but especially *P. falciparum*, could bolster this probability through evasion or suppression of host immune responses against the immature gametocytes. However, if the long term lingering of mature gametocytes at low density in the host is also important to ensure transmission, then evasion or suppression of antibodies against the mature stages would bolster probability of transmission as well.

## Background

Although the pathology of malaria is mostly due to asexually reproducing forms of the *Plasmodium* parasites within their hosts [1-3], these forms cannot survive in the mosquito vectors of the diseases. Instead, by a process not fully understood [4,5], specialized sexual forms called gametocytes arise within the host, as progeny of asexual forms, and it is these cells which propagate the infection. The mating gametes emerge within the midgut of the mosquito from gametocytes harbored in the blood meal.

The resultant zygote becomes an ookinete and then an oocyst that produces thousands of sporozoites which invade the salivary glands of the vector and thereby carry the infection to a new host when the mosquito feeds [6-8]. Since *Plasmodium* species infect vertebrates, gametocytes along with the asexual intrahost forms have to contend with vertebrate immune responses. Many studies have shown that antibodies elicited by intrahost stages of the parasite can interfere with the mating of the gametes in the bolus of the blood meal in the mosquito gut, thus blocking transmission of the pathogen. Production of such antibodies (“reaching” from the host into the vector) has been demonstrated during *Plasmodium* infections in chickens [9,10], mice [11], Rhesus monkeys [12], and human infections with *Plasmodium falciparum*[13] and *Plasmodium vivax*[14,15]. Other studies have shown that monoclonal antibodies against specific surface antigens on the gametes of *P. falciparum* can reduce the number of oocysts in mosquitoes [16,17].

The effects of a host’s acquired immunity upon the intrahost gametocytes before uptake by mosquitoes are less clear. A 1977 study [18] of eleven Gambian children who carried *P. falciparum* gametocytes found that four subjects had antibodies against gametocytes, but these antibodies did not interact with the surface of erythrocytes parasitized by gametocytes. The seven subjects without antibodies to gametocytes still were able to eliminate them. Since this study, though, newer evidence suggests a reconsideration of transmission blocking due to acquired immunity to eliminate or otherwise cripple gametocytes before uptake by the vector [19]. In particular a 2008 study of Gambian children showed that 34% of them had antibodies to antigens on the surface of erythrocytes parasitized with mature gametocytes (referred to gametocyte surface antigens or GSAs) of the 3D7 strain of *P. falciparum*[20]. This study also showed that those patients treated with chloroquine and/or sulphadoxine-pyrimethamine for four weeks had statistically significantly lower gametocyte density after treatment if they had antibodies to GSAs than if they did not. (Chloroquine and sulphadoxine-pyrimethamine act against asexual forms, and do not block gametocytemia in patients infected with *P. falciparum* parasites sensitive to these drugs [21]).

In addition to activating acquired immune responses, gametocytes, along with other intrahost stages, interact with host innate immune responses as well [22]. Studies of *Plasmodium cynomolgi* infections in toque monkeys showed that cytokines TNF and INF- *γ* were needed for the killing of gametocytes [23]. Another experiment demonstrated that white blood cells need nitric oxide to kill gametocytes of *P. falciparum* and *P. vivax*[24]. A recent study on mice infected with *Plasmodium chabaudi* showed that blockage of the TNF receptor (by antibodies to this receptor) increased the transmissibility of this parasite [25]. Finally, epidemiological evidence suggest species-dependent differences in human immune responses. Gametocytemia was found to correlate with high fever in *P. vivax* infection, but not in *P. falciparum* infection, in two studies: one of malaria patients in Peru and Thailand [26], and another of neurosyphilis patients undergoing malariatherapy [27]. Thus, gametocyte-host immune system interactions remain an active area of research for many reasons.

**Figure 1:**
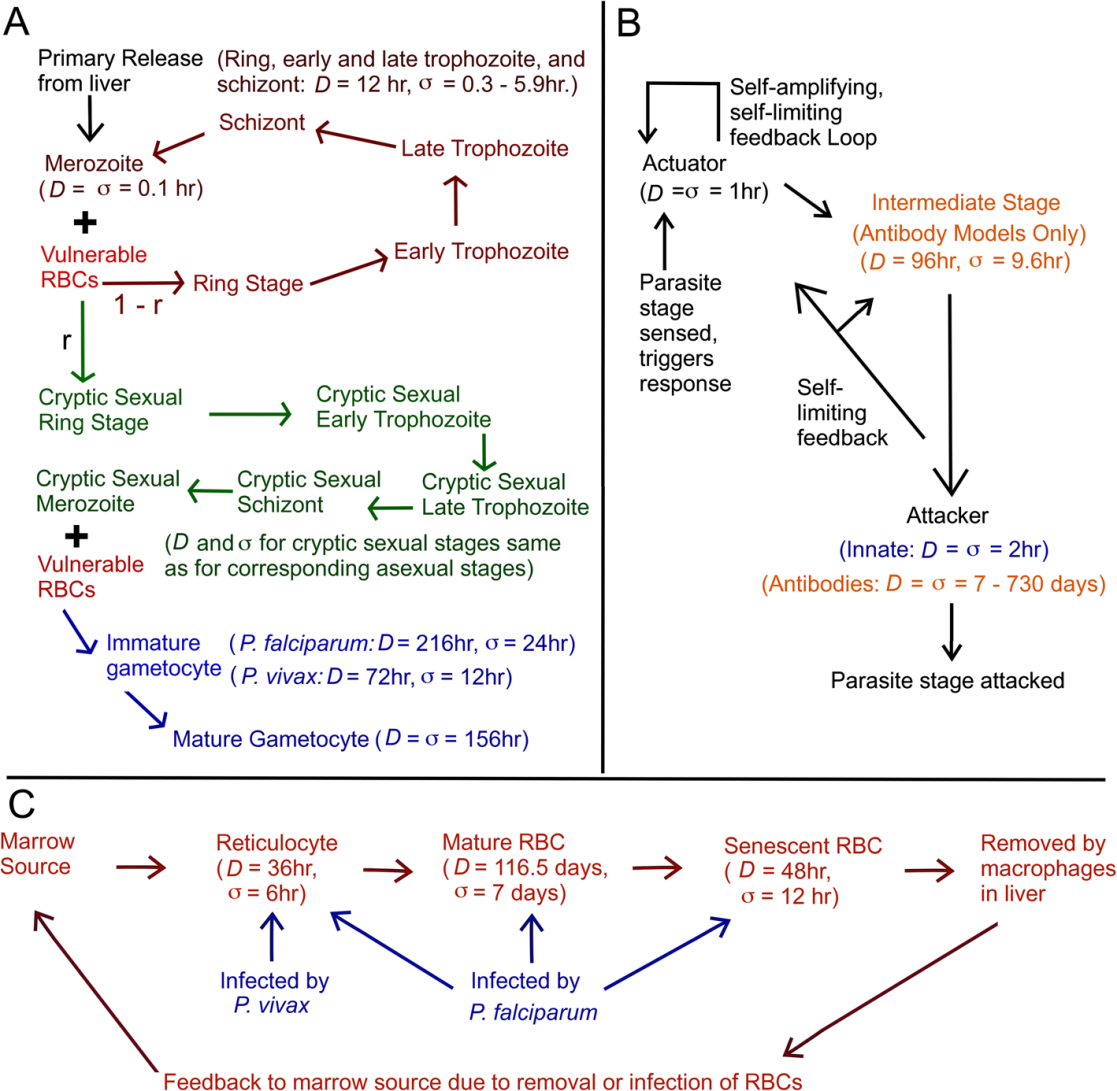
**Schematic of the cell population models.** (**A**) parasite stages. (**B**) Host immunity mechanisms. (Strictly speaking, the host immunity models are not those of cell populations, but the dynamics are similar and controlled by host cell systems.) (**C**) Erythrocyte stages. For each population phenotype, *D* is the population- mean duration and *σ* is the standard deviation in the duration. In (**A**), *r* is the proportion of asexual merozoites which are diverted down the pathway of gametocyte development. Interactions between cell phenotypes are explained in depth in the Methods, as well as description of model parameters.

Although host responses to *Plasmodium* infection are extremely complex, one can apply the logic of computation to determine the infection and transmission outcome for a hypothetical set of host responses if one suspects those responses to be important for modulating the course of infection. For this report, ideas from population biology were used to build mathematical models of the dynamics of (1) the gametocyte population, (2) the population of the asexual stages that give rise to the sexual forms, and (3) the population of red blood cells of the host that sustain the parasite. Different modalities of immune responses to both the asexual parasites and gametocytes are incorporated, as well as varying degrees of dyserythropoiesis, which is often observed in malaria infections [28]. Immune responses and erythropoietic responses have their own dynamics. The models used here are based on ones previously developed by the authors to study other aspects of intra-host *Plasmodium* dynamics [29-31]. The models consist of coupled ordinary differential equations which were chosen to allow for flexibility to incorporate both interaction between populations and varying temporal scales of processes that affect the populations. The formalism is development in the Methods section below. Figure 1 shows schematics of the population models. Each population is parameterized by *D*, the average duration of an individual member of the population, and *σ*, the standard of deviation in the durations of individuals. Note that a “cryptic sexual” (CS) model of gametocytogenesis is assumed: experiments conducted in cultures of *P. falciparum* in human blood indicate that some schizonts have progeny that are primary sexual progeny, while progeny of other schizonts are primarily asexuals [32,33]. Gene expression work on cultured *P. falciparum* indicates that some early ring stage parasites [34] and schizonts [35] are apparently committed to having primarily sexual progeny. Two reports suggest that a schizont committed to producing gametocytes has either male or female progeny [36,37]. For simulation results reported here, it is assumed that 5% of the merozoites from a bursting asexual schizont become committed to forms that have sexual progeny; (that is, *r*=0.05 in Figure 1). Committed forms in this report are called “cryptic” because they otherwise resemble the asexual intraerythrocytic forms.

Since much about gametocytogenesis is still uncertain, a “non-cryptic sexual” (non-CS) modality of gametocytogenesis was examined as a test of robustness of the model results. In this modality 5% of merozoites from each bursting schizont go directly into gametocyte development after parasitizing an erythrocyte with no cryptic sexual forms. Results for models that incorporated the non-CS modality were found to be very similar to results form simulations using the CS modality. For that reason, this report focuses on results from models using the CS modality, while results based on non-CS models are given in Additional file 1.

The two major *Plasmodium* species responsible for the bulk of public health burden of human malarias are *P. falciparum* and *P. vivax*[3,38], which share the same basic lifecycle and development stages. Gametocytogenesis during *P. falciparum* infection apparently is modulated by factors in the blood as infection progresses [39-42]; for simplicity it is assumed that commitment of some merozoites to the sexual pathway starts when the density of intraerythrocytic asexual forms reaches 0.01 *μL*^−1^ for both species. Despite the similarities of *P. falciparum* and *P. vivax*, their last common ancestor was ≈10^8^ years ago [43,44], so they have evolved major physiological differences. The mature gametocytes of the two species have different physical appearance: those of *P. falciparum* have an elongated or crescent shape, while those of *P. vivax* tend to be round [45,46]. Immature gametocytes of *P. falciparum* sequester during that period in bone marrow [47], and perhaps other organs [48] while those of *P. vivax* remain visible in the peripheral blood, (although possible sequestration of *P. vivax* in the human host remains an area of investigation) [49]. In this report, the two major differences of interest are the time required for gametocytes to reach maturity, and age specificity of the erythrocytes attacked. The two species differ in the time required for gametocytes to mature into the forms that can be taken up by mosquitoes: gametocytes of *P. vivax* mature in 2-4 days, while those of *P. falciparum* require 6-10 days [50,51]. *P. vivax* invades mainly the very youngest RBCs, the reticulocytes, while *P. falciparum* can attack RBCs of any age [52-54]. Thus, the mathematical models were tailored to take into account these differences between *P. vivax* and *P. falciparum*. It will be seen that the difference in maturity time can lead to very different gametocyte population dynamics for the two species. The focus of the Results and discussion section is on the characteristic effects host immune responses have upon the population dynamics of transmissible gametocytes as predicted by the model formalism.

## Results and discussion

The outcomes of simulated malaria infections were determined for thousands of values of model parameters, allowing us to examine a large spectrum of behavior. All simulations incorporated the following two host immune responses: (1) a quickly acting, quickly decaying innate-like response that is triggered by merozoite production and acts against all the asexual intracellular parasites and their cryptic sexual counterparts (a “pyrogenic” response), and (2) a slowly acting, slowly decaying response to emulate an antibody response triggered by and acting against schizonts. For each immune responses, including any against gametocytes, the parasitemia levels that trigger the response and the maximum rates of target clearance were parameters varied from simulation to simulation; see “Simulating immune response dynamics” in Methods below for details.

Severe and potentially fatal cases of both *P. falciparum* malaria [2] and *P. vivax* malaria [55] manifest as respiratory distress, cerebral complications, severe anemia, and other symptoms, but for this report it is assumed that the host dies if the uninfected red blood cell counts drops below 60% of normal, either by direct parasitization of erythrocytes or dyserythropoiesis or both. This reports gives results only for simulated infections for which intracellular asexual forms exceed the threshold to trigger sexual forms (0.01 *μL*^−1^), and infection duration is less than three years.(Here, infection duration means the time from primary release of merozoites from the liver until either the clearance of all forms of the parasite from the host or death of host.) Over 65% of the simulations generated met these criteria. Of those that did, ≈5*%* resulted in death of host, with a median survival time of 152 days for *P. falciparum* and 161 days for *P. vivax*. Of the simulations considered for this report in which the host clears the parasite, the duration of the asexual forms in the host ranged from 2 days to over 1080 days, for both *P. falciparum* and *P. vivax* with a median ≈11 days for both species. The results of over 10^5^ simulations were used for this report. Immune constraints on the asexual parasite population dynamics were discussed in a previous publication [30], so this report focuses mainly on the behavior of the gametocytes. (Additional file 1: Figures S1–S5 give a summary of the behavior of the asexual populations as a function of model parameters.)

### Example time series

**Figure 2:**
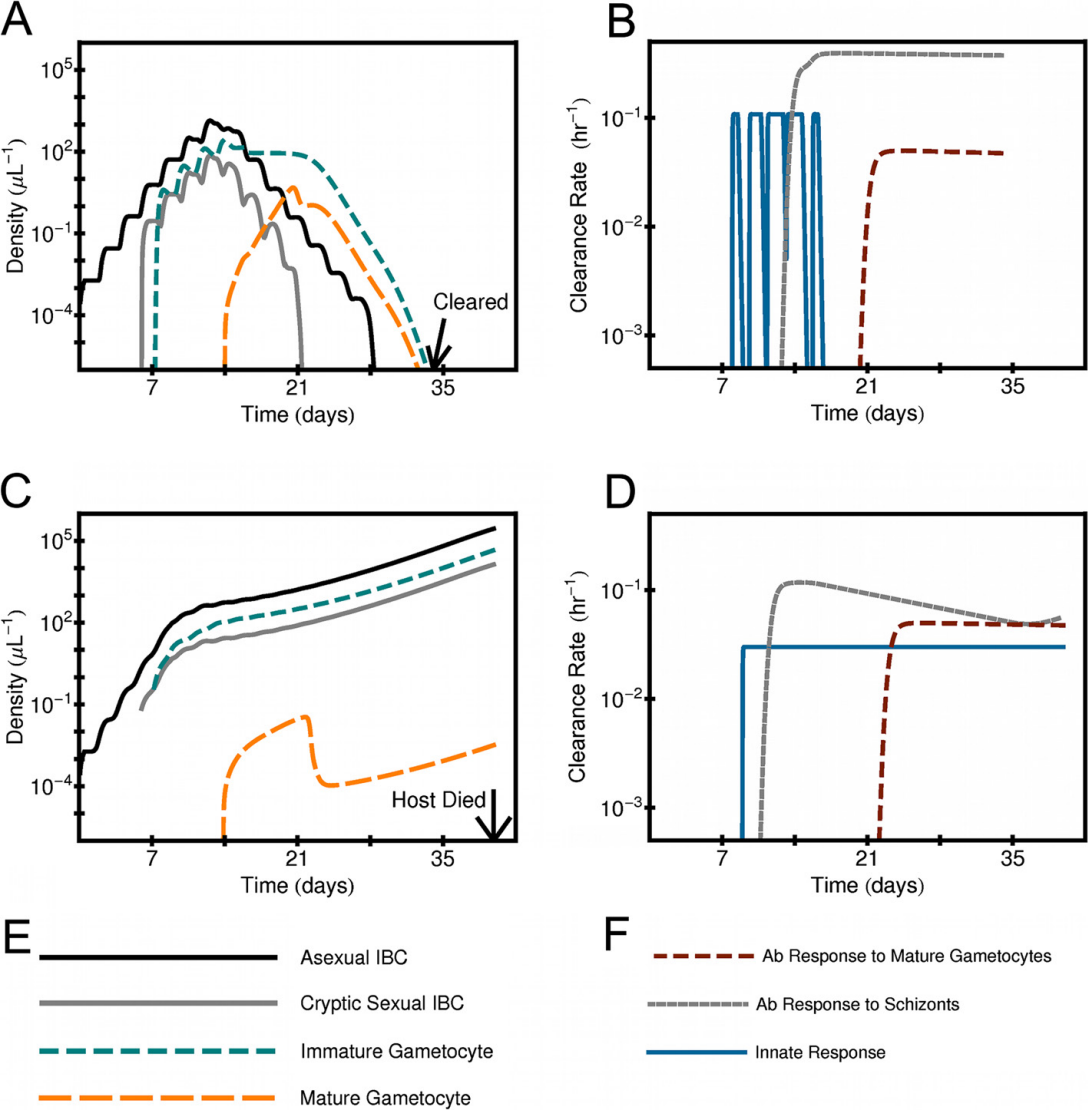
**Time series of parasite density for two simulated infections with*P. falciparum*.** (**A**) Blood density of different stages of the parasite as a function of time in an infection for which the host clears all parasite forms. (**B**) Clearance rate of various immune responses as a function of time since primary release, same infection as (**A**). (**C**) Blood density of different stages of the parasite as a function of time in an infection for which the host dies of anemia. (**D**) Clearance rate of various host immune responses as a function of time since primary release, same infection as (**C**). (**E**) Color code used for (**A**) and (**C**) for parasite forms. (**F**) Color code used for (**B**) and (**D**) for host immune modalities. The initial time is at the start of primary release of merozoites from the liver. The model parameters used for these two examples are given in Table 1.

Figure 2 shows example time series of parasitemia and rate of target clearance of immune effectors for two simulated infections with *P. falciparum* in which host immune responses attack mature gametocytes (as well as other targets). The host clears all parasite forms in one infection, (illustrated in panels 2A and 2B), and in the other the host immune responses fail to control asexual parasitemia and the host dies of anemia (panels 2C and 2D). Table 1 gives the values of the model parameters used for the two simulations. These two examples were chosen to illustrate that the simulated infections have non-trivial population dynamics resembling those in real infections, although these two are not meant to be the most representative nor realistic of all the simulations. However, these two examples illustrate the following: (1) mature gametocytes can linger after all the asexual intracellular forms are cleared in a non-lethal infection. (2) If the gametocytes are vulnerable to host immune responses, those responses can suppress the density of the mature gametocytes despite an overall large parasitemia. Both the lingering of gametocytes and their suppression by host immune responses are discussed in more detail below.

**Table 1:** **Parameters for the two simulated infections shown in Figure**2

**Parameter**	**Value for cleared infection**	**Value of fatal infection**
*σ*_*Asx*_	0.782493 *hr*	3.28359 *hr*
*δ*_*Dys*_	1.70384	4.87089
*μ*_*Th*_	0.0192832 *μL*^−1^	0.0396408 *μL*^−1^
*χ*_*Inn*,*Mx*_	0.566114 *hr*^−1^	0.630405 *hr*^−1^
*Sc*_*Th*_	9.53473 *μL*^−1^	1.18172 *μL*^−1^
*χ*_*Abc*,*Mx*_	0.438998 *hr*^−1^	0.0559842 *hr*^−1^
$\lambda_{\chi_{\text{Ab},\text{Sc}}}$	7904.34 *hr*	548.325 *hr*
*IG*_*Th*_	*∞*	*∞*
*χ*_*AbG*,*Mx*_	0	0
$\lambda_{\chi_{\text{Ab},\text{IG}}}$	0	0
*MG*_*Th*_	0.0385589 *μL*^−1^	0.0110744 *μL*^−1^
*χ*_*Ab*,*MG*,*Mx*_	1.20388 *hr*^−1^	27.1358 *hr*^−1^
$\lambda_{\chi_{\text{Ab},\text{MG}}}$	4136.27 *hr*	6560.52 *hr*

Both infections were with *P. falciparum* using the CS model for gametocytogenesis. The model parameters are defined in Methods.

### Model results if gametocytes are invisible to acquired immunity

**Figure 3:**
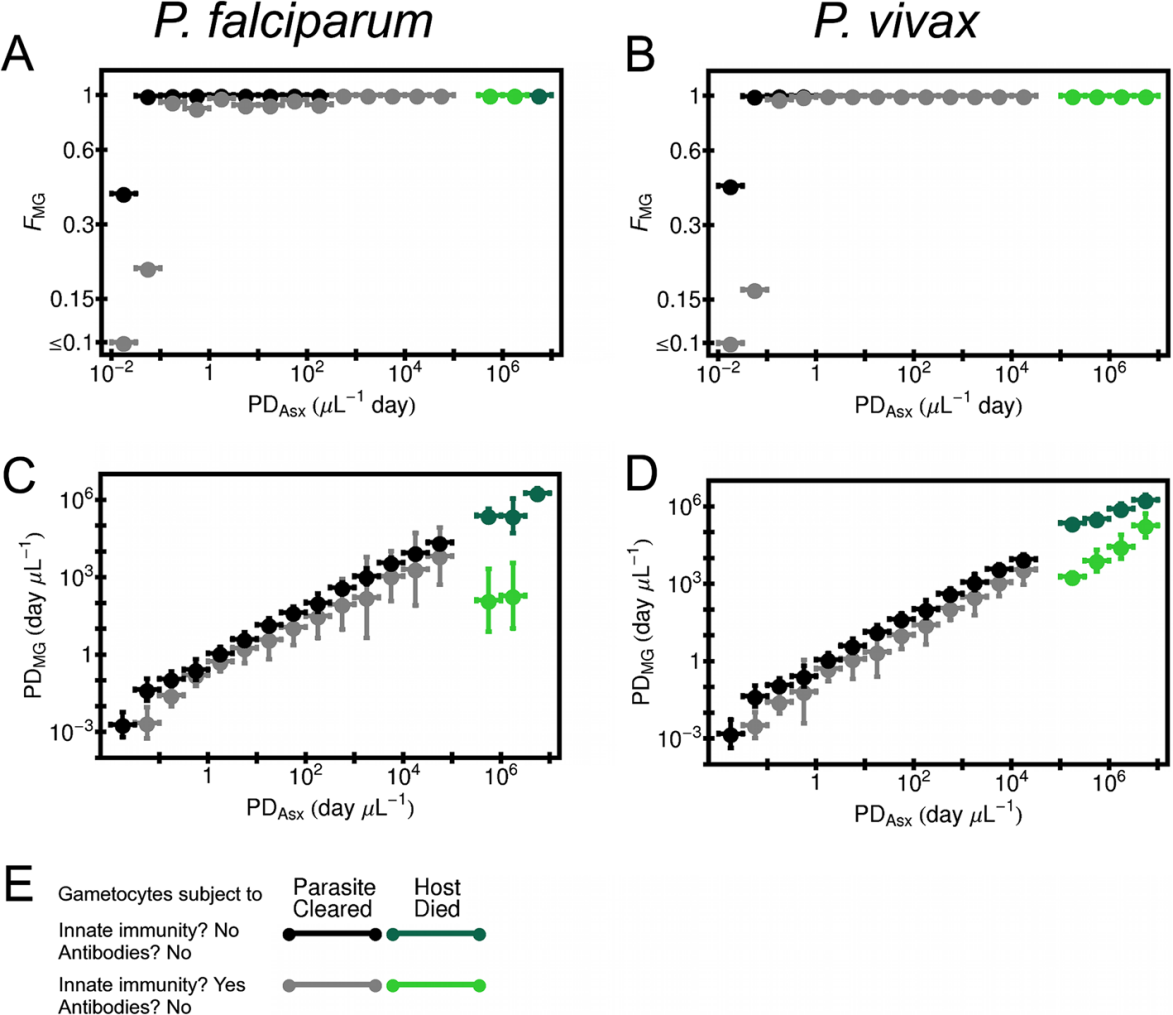
**Efficiency of mature gametocytes production versus host exposure to asexual forms if gametocytes unaffected by acquired immunity.** Both immature and mature gametocytes are unaffected by the host’s acquired immunity. The simulations were binned by the logarithms of the parasite-days of intracellular asexual forms, *PD*_*Asx*_; see text for definition. The horizontal bars show the bin width.(**A**) Fraction of simulations making mature gametocytes *F*_*MG*_ versus *PD*_*Asx*_ for simulated *P. falciparum* infections. (**B**) Same as (**A**), except for simulated *P. vivax* infections. (**C**) Logarithmic average of mature gametocyte parasite-days *PD*_*MG*_ versus *PD*_*Asx*_ for those simulated *P. falciparum* infections in which mature gametocytes are produced. Vertical bars are the standard deviation in the logarithmic average. (**D**) Same as (**C**) except for simulated *P. vivax* infections. (**E**) Color code for the host immunity-gamteocyte models. “Parasite Cleared”: host cleared all parasite forms within three years after primary release of merozoites from the liver. “Host Died”: host died of anemia within three years of primary release. Note: for each of two immune classes and host fates color coded, results are shown only for bins which contained 10 or more simulations.

Before considering the effects of acquired immunity directed directly against gametocytes, it is useful to look at the behavior of model infections if gametocytes are not under any direct pressure from acquired immunity. Then the host’s acquired immune responses can affect gametocytes only by eliminating asexual and cryptic sexual forms before they can produce gametocytes. Figure 3 shows the results for the production of the transmissible (mature) gametocytes in this case. In the figure, the quantity “asexual parasite days,” *PD*_*Asx*_, defined as the average density in the blood of the asexual intracellular parasitic forms during infection times the duration of infection, is used as an index of the burden upon the host due to the asexual forms. The measures of gametocytemia are shown in Figure 3, (1) the fraction of simulated infections in which in mature gametocytes are produced, *F*_*MG*_, and (2) mature gametocyte parasite-days, *PD*_*MG*_, defined as the average blood density of the mature gametocytes times the duration of infection. The values shown for *F*_*MG*_ and *PD*_*MG*_ in Figure 3 are ensemble averages over simulations for which *PD*_*Asx*_ has been grouped into bins. (In the case of *PD*_*MG*_, it is the geometric mean.) The sampling of the parameter spaces introduces stochastic fluctuations within the bins of *PD*_*Asx*_. (See “Sampling the parameter space” in the Methods.) If the gametocytes are not removed by the model innate immunity response, then almost all simulated infections made mature forms unless *PD*_*Asx*_≪1 *μL*^−1^*day* (panels 3A and 3B). Furthermore, for the subset of these simulations that produce mature gametocytes, the value of *PD*_*MG*_ for both *Plasmodium* species is correlated almost linearly to *PD*_*Asx*_: for *P. falciparum*, $PD_{\text{MG}}\approx 0.5\times PD_{\text{Asx}}^{1.03}$, and for *P. vivax*, $PD_{\text{MG}}\approx 0.48\times PD_{\text{Asx}}^{1.05}$. (See panels 3C and 3D.) Since the mature gametocytes can linger in the host for weeks after the asexuals are cleared, the slope of ≈0.5 is much larger than 0.05, the conversion ratio *r* of asexuals to sexuals.

If the model innate response can remove gametocytes, almost all simulated *P. vivax* infections still make mature gametocytes if *PD*_*Asx*_≥ 1 *μL*^−1^*day*, although there is some reduction in *F*_*MG*_ for *P. falciparum* infections if *PD*_*Asx*_≥ 1 *μL*^−1^*day*. For the subset of simulated infections that make mature gametocytes, the range in *PD*_*MG*_ for a given *PD*_*Asx*_ is somewhat larger, but *PD*_*MG*_ still grows almost linearly with *PD*_*Asx*_, although at a reduced level: for *P. falciparum*, $PD_{\text{MG}}\approx 0.17\times PD_{\text{Asx}}^{0.99}$, and for *P. vivax*, $PD_{\text{MG}}\approx 0.14\times PD_{\text{Asx}}^{1.05}$. The exception is for when the host dies, especially for *P. falciparum*: then *PD*_*MG*_ is suppressed by one to three orders of magnitude if the innate response affects gametocytes (versus when it does not). Investigation of model infections that end in death of host showed that the innate immune response is triggered to be active most of the time due to the failure of innate and acquired immunity combined to control the asexual forms (as for the example illustrated in Figure 2C and 2D). A useful quantity to quantify the activeness of the innate immune response is the time integral of the clearance rate of the innate response integrated over the course of infection, *Iχ*_*Inn*_. For the *P. falciparum* simulations that ended in host death, *Iχ*_*Inn*_=108.4, while *Iχ*_*Inn*_=15.0 for simulations in which the parasite is cleared with *PD*_*Asx*_ in the interval 10^4.5^*μL*^−1^*day*−10^5^*μL*^−1^*day*. (Note that *Iχ*_*Inn*_ is a dimensionless quantity.) Similarly, for the *P. vivax* simulations that ended in host death, *Iχ*_*Inn*_ = 125.6, while *Iχ*_*Inn*_=4.53 for simulations in which the parasite is cleared with *PD*_*Asx*_ in the interval 10^4^*μL*^−1^*day*−10^4.5^*μL*^−1^*day*. Since gametocytes for *P. falciparum* and *P. vivax* have a longer duration for development than the asexual stages, the gametocytes would be vulnerable if a sustained immune response could affect them. The case of infections ending with host death is analyzed in more detail in the Discussion section below.

**Figure 4:**
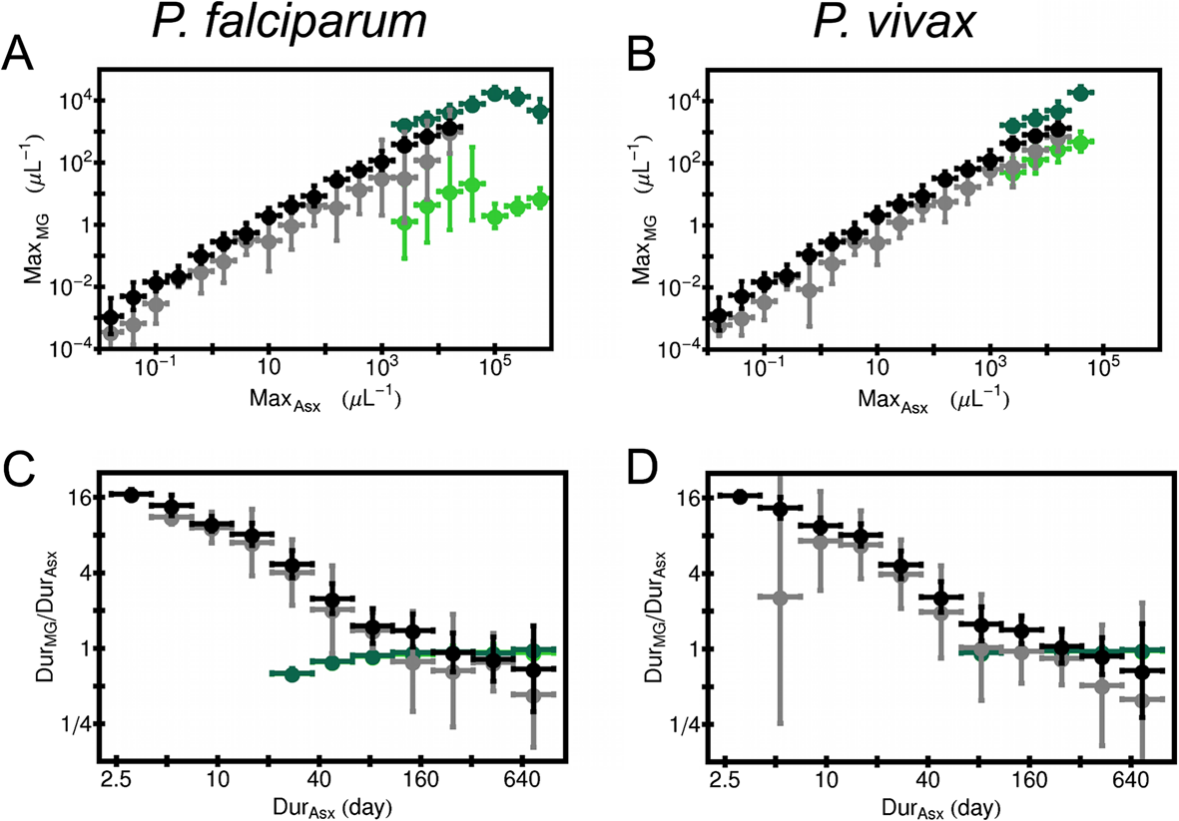
**Some clinically observable properties of mature gametocytes if gametocytes unaffected by acquired immunity.** Immature and mature gametocytes unaffected by host acquired immunity response. The color code used in each panel is the same as for Figure 3. (**A**) Plot of maximum blood density of mature gametocytes, *Max*_*MG*_, versus maximum density of intracellular asexual forms, *Max*_*Asx*_ for simulated *P. falciparum* infections. The simulations were binned by the logarithm of *Max*_*Asx*_, and horizontal bars show the width of each bin. The logarithmic average of *Max*_*MG*_ are computed for each bin for each of the four types of models color coded. Only simulations that produced mature gametocytes are included, and results are only shown for bins that contained 10 or more simulations. (**B**) Same as (**A**), except for simulated *P. vivax* infections. (**C**) Plot of ratio of duration of mature gametocytes in the host *Dur*_*MG*_ to duration of asexual forms *Dur*_*Asx*_ versus *Dur*_*Asx*_ for simulated *P. falciparum* infections. The simulations were binned by the logarithm of *Dur*_*Asx*_, and horizontal bars show the width of each bin. The logarithmic average of *Dur*_*MG*_/*Dur*_*Asx*_ are computed for each bin for each of the four types of models color coded. Only simulations that produced mature gametocytes are included, and results are only shown for bins that contained 10 or more simulations. (**D**) Same as (**C**), except for simulated *P. vivax* infections.

Clinical observation of a patient would track the density of parasite stages in the blood directly, rather than *PD*_*Asx*_ or *PD*_*MG*_, so shown in Figure 4 is the maximum blood density of mature gametocytes, *Max*_*MG*_, as a function of maximum blood density of the asexual forms, *Max*_*Asx*_, for simulated infection in which the gametocytes are invisible to acquired immunity and mature forms are made. The density *Max*_*MG*_ tracks *Max*_*Asx*_ almost linearly for those infections in which the host’s immune responses clear the parasites Just as *PD*_*MG*_ is suppressed if the gametocytes are vulnerable to the model innate response, *Max*_*MG*_ is also in this case. *Max*_*MG*_ is especially suppressed in *P. falciparum* model infections that end in host death. Also shown in Figure 4 is the ratio of the duration of the mature gametocytes in the blood, *Dur*_*MG*_, to the duration of the asexual forms, *Dur*_*Asx*_, as a function of *Dur*_*Asx*_ for the same set of simulations. It is assumed that without any immune pressure, the mature gametocyte population would decay away with a time constant *D*_*MG*_=156 *hr*[56], as discussed more in Subsections “Model for population dynamics of sexual forms”. Thus, in this model mature gametocytes may linger for weeks in model infections even if the asexual forms are cleared by the host’s immune responses within a few days.

Similar results were found for simulations with the non-CS models; see Additional file 1: Figures S6 and S7.

### Antibodies against immature gametoctyes tend to be more effective in reducing the density of transmissible gametocytes than antibodies directly against the transmissible forms

Two modalities of acquired response against gametocytes were considered: (1) an antibody response to the mature gametocytes, and (2) an antibody response to the immature gametocytes. It is assumed that the vulnerable stage makes the triggering antigen during the entire duration of the stage. As a measure of the strength of the antibody response to the gametocyte, the quantity *IS*_*Gcy*_=*χ*_*Ab*,*Mx*_/*Tar*_*Th*_ was defined, where *χ*_*Ab*,*Mx*_ is the maximum clearance rate of the targeted stage, and *Tar*_*Th*_ is the density of the targeted stage which triggers the response. See “Model for immune response dynamics” in the Methods section below.

**Figure 5:**
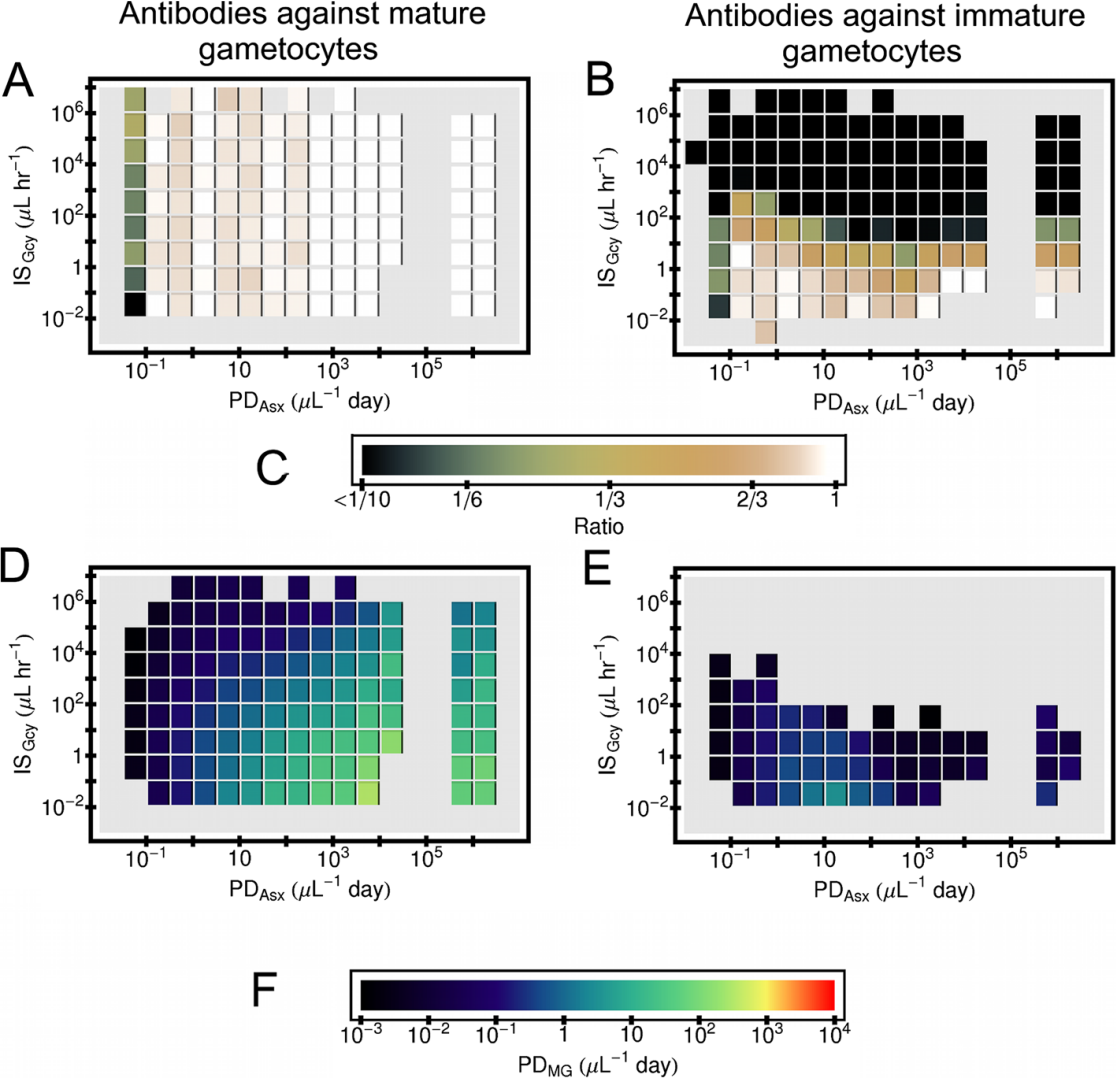
**Effects of gametocyte-specific antibodies in simulated*P. falciparum* infection on gametocyte levels.** Results from simulated *P. falciparum* infections that resolved within three years from primary release of merozoites were binned by parasite-days of the intracellular asexual forms, *PD*_*Asx*_, and also by a measure of the ability of the antibody response to clear their target, *IS*_*Gcy*_. See text for definitions. The horizontal length of an individual block shows the bin size in *PD*_*Asx*_, and the vertical extent of a block show the bin size in *IS*_*Gcy*_. Results are only shown for bins that contained 10 or more simulations. (**A**) Fraction of simulations making mature gametocytes if a model antibody response attacks mature gametocytes. (**B**) Same as (**A**), except a model antibody response attacks immature gametocytes. (**C**) Color code for panels (**A**) and (**B**). (**D**) Logarithm mean of parasite days of mature gametocytes *PD*_*MG*_ for simulated infections producing mature gametocytes if a model antibody response attacks mature gametocytes. (**E**) Same of (**D**) except a model antibody response attacks immature gametocytes. (**F**) Color code for panels (**D**) and (**E**). Note: host died in all simulate infections for which *PD*_*Asx*_> 10^5^*μL*^−1^*day*, and parasite was cleared in all simulated infections if *PD*_*Asx*_<10^5^*μL*^−1^*day*.

The plots in Figure 5 show the effects of these two modalities of host acquired immunity against gametocytes on the production of the mature gametocytes for the simulated *P. falciparum* infections. (For all the simulated infections considered for these plots, the model innate response is also active.) The fraction of simulations making mature gametocytes, *F*_*MG*_ and the mature gametocyte parasite-days, *PD*_*MG*_ are shown as functions of two quantities, asexual parasite days *PD*_*Asx*_, and *IS*_*Gcy*_. If antibodies acting directly on mature gametocytes are present, they should not affect whether or not an infection generates mature forms, and indeed for this immune modality *F*_*MG*_ essentially depends only on *PD*_*Asx*_; see panel 5A. (Remember that the value shown for each tile in panels of Figure 5 is an ensemble average of results of several simulations.) However, if the antibody attacks immature gametocytes instead, then the larger *IS*_*Gcy*_ is, the smaller *F*_*MG*_. In fact, production of the transmissible forms of *P. falciparum* is then almost completely suppressed in a large part of the model parameter space even for the largest values of *PD*_*Asx*_; see panel 5B. In addition, for a given *PD*_*Asx*_ and *IS*_*Gcy*_, *PD*_*MG*_ is suppressed more, on average, if antibodies act against the immature gametocytes rather than the mature forms; see panels 5D and 5E. In Additional file 2 there is an explanation of why this might be the case using simplified version of the acquired immunity models.

**Figure 6:**
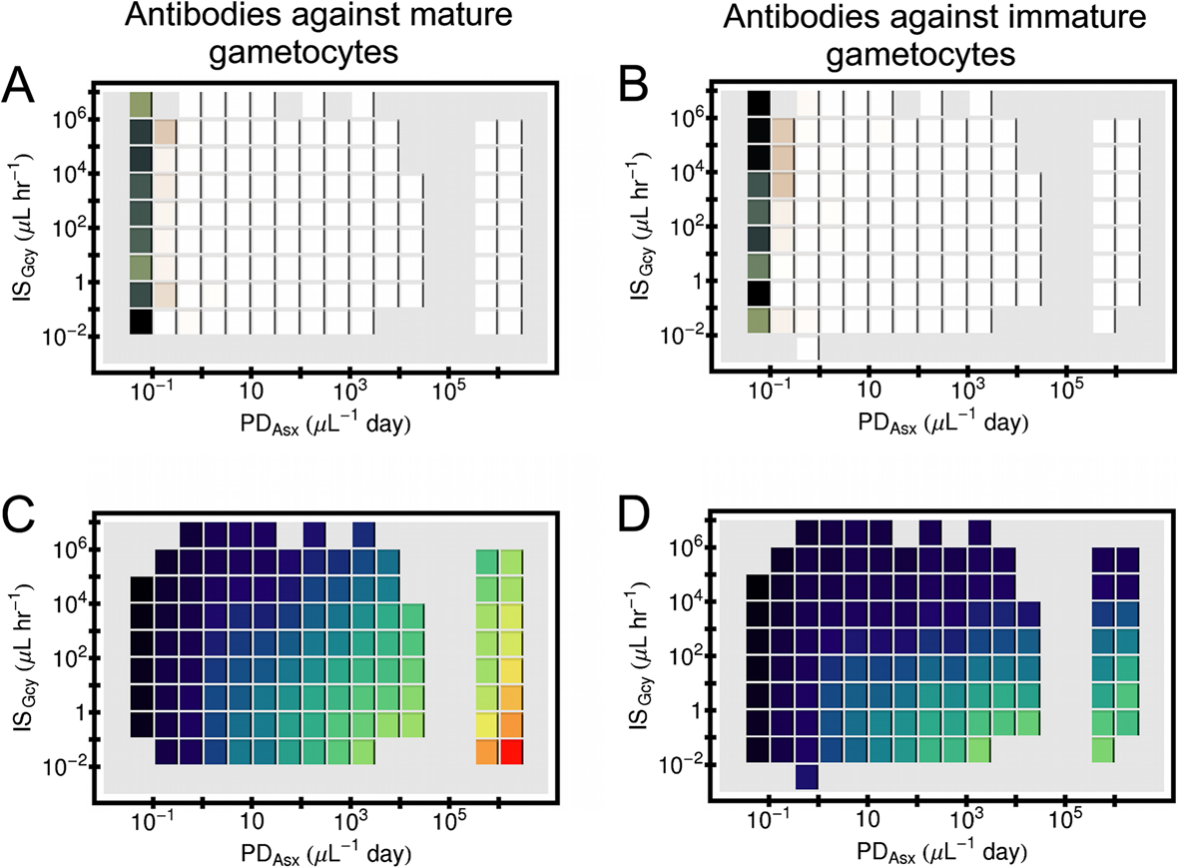
**Effects of gametocyte-specific antibodies in simulated*P. vivax* infection on gametocyte levels.** Results from simulated *P. vivax* infections that resolved within three years from primary release of merozoites were binned by parasite-days of the intracellular asexual forms, *PD*_*Asx*_, and also by a measure of the ability of the antibody response to clear their target, *IS*_*Gcy*_. See text for definitions. The horizontal length of an individual block shows the bin size in *PD*_*Asx*_, and the vertical extent of a block show the bin size in *IS*_*Gcy*_. Results are only shown for bins that contained 10 or more simulations. (**A**) Fraction of simulations making mature gametocytes if a model antibody response attacks mature gametocytes. (**B**) Same as (**A**), except a model antibody response attacks immature gametocytes. Color code for panels (**A**) and (**B**) is the same as for panels (**A**) and (B) in Figure 5. (**C**) Logarithm mean of parasite days of mature gametocytes *PD*_*MG*_ for simulated infections producing mature gametocytes if a model antibody response attacks mature gametocytes. (**D**) Same of (**C**) except a model antibody response attacks immature gametocytes. Color code for panels (**C**) and (**D**) are the same as for panels (**D**) and (**E**) in Figure 5. Note: host died in all simulate infections for which *PD*_*Asx*_> 10^5^*μL*^−1^*day*, and parasite was cleared in all simulated infections if *PD*_*Asx*_< 10^5^*μL*^−1^*day*.

The duration of the immature gametocyte phase of *P. vivax* is ≈1/3 that of *P. falciparum*, so most of the simulated *P. vivax* infections will make mature gametocytes, even if the immature gametocytes are attacked by a model antibody response; see panels A and B in Figure 6. However, for a given *PD*_*Asx*_ and *IS*_*Gcy*_, *PD*_*MG*_ is still suppressed more on average if antibodies act against the immature gametocytes than against the mature forms, as shown in panels C and D in Figure 6.

For the model antibody modalities, the non-CS models showed very similar behavior; see Additional file 1: Figures S8 and S9.

### When infections make mature gametocytes, antibodies against mature gametocytes are more effective in reducing the window of transmission than antibodies against the immature forms

**Figure 7:**
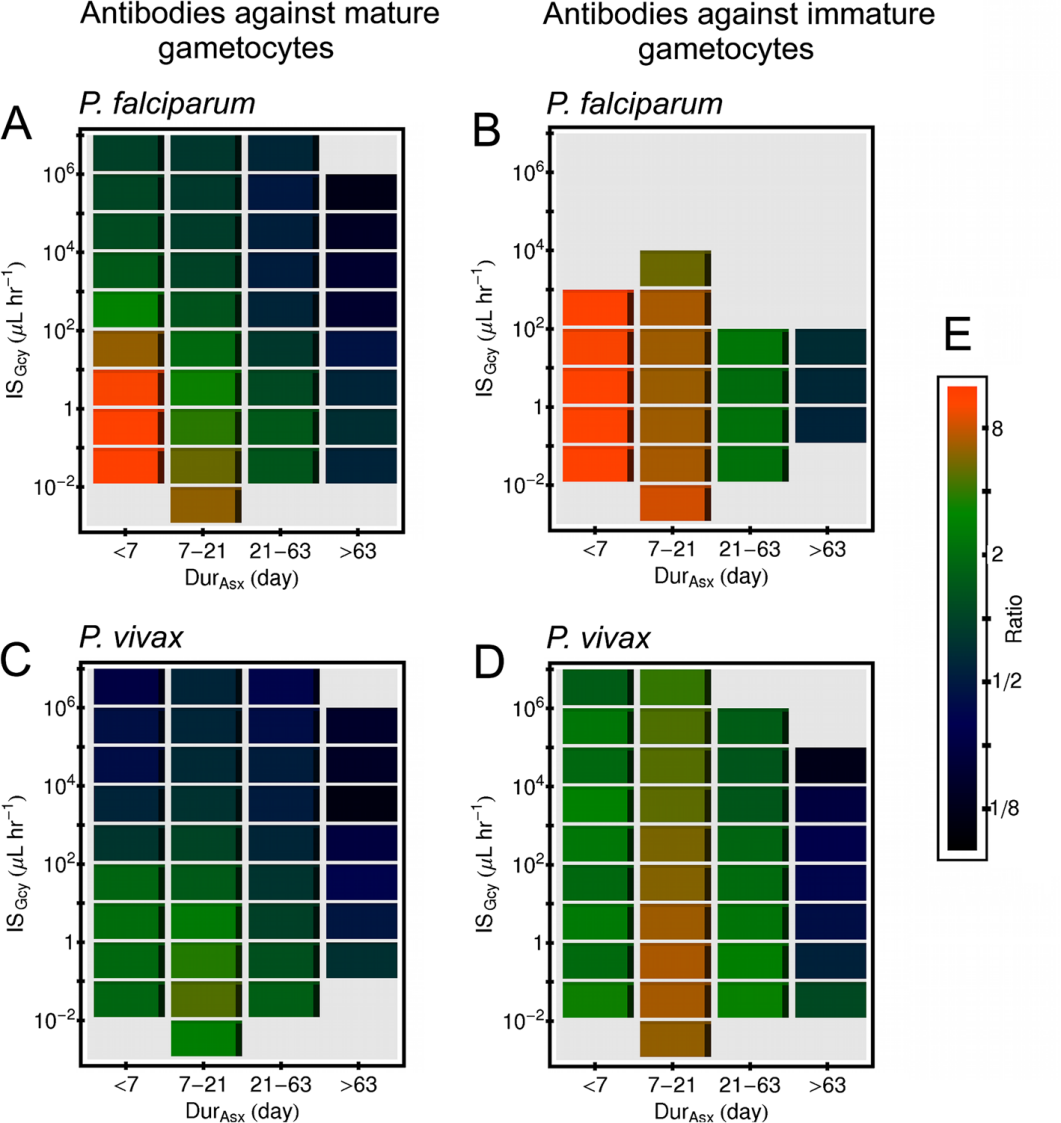
**Effects of gametocyte-specific antibodies in simulated infection on gametocyte duration.** Results are from simulated infections in which the host cleared all parasites within three years from primary release of merozoites, and which also made mature gametocytes. The ratio of the duration of mature gametocytes, *Dur*_*MG*_ to the duration of the asexual forms, *Dur*_*Asx*_ was binned by *Dur*_*Asx*_, and also by a measure of the ability of the antibody response to clear their target, *IS*_*Gcy*_. The horizontal length of an individual block shows the bin size in *Dur*_*Asx*_, and the vertical extent of a block show the bin size in *IS*_*Gcy*_. Results are only shown for bins that contained 10 or more simulations. (**A**) *Dur*_*MG*_/*Dur*_*Asx*_ for simulated *P. falciparum* infections in which antibodies attacked mature gametocytes and not immature forms. (**B**) *Dur*_*MG*_/*Dur*_*Asx*_ for simulated *P. falciparum* infections in which antibodies attacked immature gametocytes and not mature forms. (**C**) Same as (**A**), except for simulated *P. vivax* infections. (**D**) Same as (**B**), except for simulated *P. vivax* infections. (**E**) Color code used for *Dur*_*MG*_/*Dur*_*Asx*_.

Now consider how antibodies against gametocytes affect the duration that mature gametocytes will be present in the host. The plots in Figure 7 show the ratio *Dur*_*MG*_/*Dur*_*Asx*_ as a function of both *Dur*_*Asx*_ and *IS*_*Gcy*_. (Again, the result in each tile is an geometric mean over an ensemble of simulations.) One can see that on average for the same values of *Dur*_*Asx*_ and *IS*_*Gcy*_ for those simulated infections that produce mature gametocytes, antibodies directly to the mature forms tend to suppress *Dur*_*MG*_/*Dur*_*Asx*_ more than antibodies against the immature forms, especially for *Dur*_*Asx*_≤ 63 days. This is true for both *P. falciparum* (panels 7A and 7B) and *P. vivax* (panels 7C and 7D). Analogous results for simulated Non-CS infections are shown in Figure S10 in Additional file 1, which is very similar to Figure 5.

### Mature gametocyte production is affected by the standard deviation in the development time of the asexual forms

**Figure 8:**
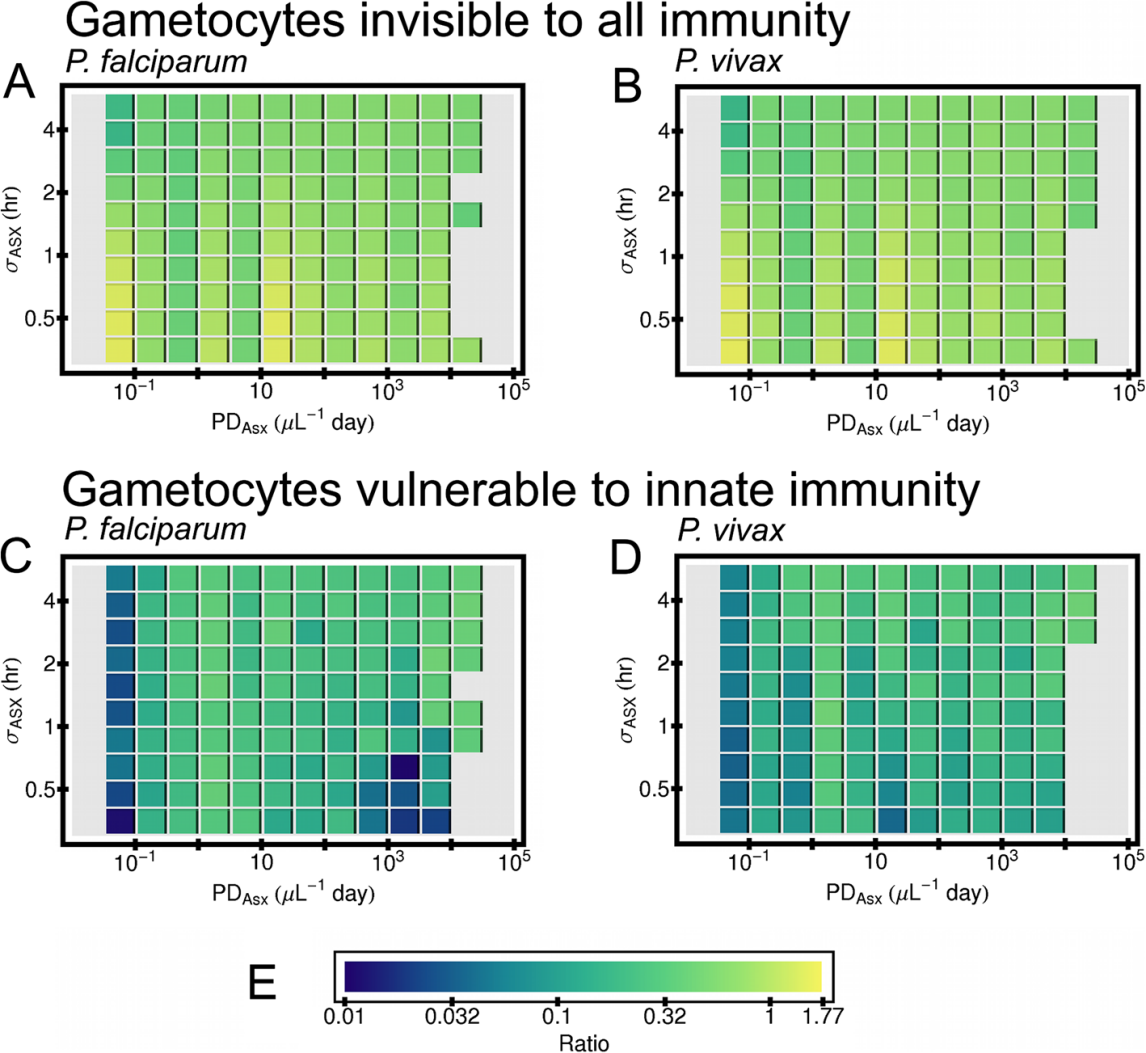
**Effects of standard deviation of development time of intracellular asexual forms on gametocyte levels.** Results are from simulated infections in which the host cleared all parasites within three years from primary release of merozoites, and which also made mature gametocytes. Gametocytes are assumed to be invisible to acquired immunity. The ratio of the parasite days of mature gametocytes to parasite days of asexual forms, *P*_*MG*_/*PD*_*Asx*_, was binned by *PD*_*Asx*_ and by the standard deviation of development time of intracellular asexual forms, *σ*_*Asx*_. The horizontal length of an individual block shows the bin size in *PD*_*Asx*_, and the vertical extent of a block show the bin size in *σ*_*Asx*_. Results are only shown for bins that contained 10 or more simulations. (**A**) *PD*_*MG*_/*PD*_*Asx*_ for simulated *P. falciparum* infections in which gametocytes are unaffected by host immune responses. (**B**) Same as (**A**), except for simulated *P. vivax* infections. (**C**) *PD*_*MG*_/*PD*_*Asx*_ for simulated *P. falciparum* infections in which model innate response cleared mature and immature gametocytes at the same rate that it clears asexual forms. (**D**) Same as (**C**), except for simulated *P. vivax* infections. (**E**) Color code used for *PD*_*MG*_/*PD*_*Asx*_.

As indicated in Figure 1, *σ* for the asexual and cryptic sexual populations was varied from simulation to simulation; see Subsection “Sampling the parameter space” below in the Methods. This was done because a previous modeling study showed that the density of the asexual forms tends to increase with an increase value of *σ* for their population, an effect apparently due to the interplay between the release of the schizonts and the triggering of the model innate immune reaction by the release of merzoites [30]. For that reason, some properties of the gametocyte population as a function of *σ* for the asexual (*σ*_*Asx*_) were investigated, with the results summarized in the plots of Figure 8. That figure shows the ratio *PD*_*MG*_/*PD*_*Asx*_ as a function of *σ*_*Asx*_ and *PD*_*Asx*_ for all simulated infections for which (1) the host clears all parasite forms, (2) the gametocytes are invisible to acquired immunity, and (3) mature gametocytes are made. (Again, the results in each tile is a geometric mean over an ensemble of simulations.) Results shown in panels 8A and 8B are for simulations in which gametocytes are unaffected by innate immunity, and results in panels 8C and 8D are for simulations in which gametocytes are affected by the model innate response. Note that for both *Plasmodium* species, *PD*_*MG*_/*PD*_*Asx*_ decreases slightly with increasing *σ*_*Asx*_, However, if the model innate response can remove gametocytes, the trend is the opposite. In particular, for simulated *P. falciparum* infections with *PD*_*Asx*_ ≥300 *μL*^−1^*day*, *PD*_*MG*_/*PD*_*Asx*_ is suppressed for *σ*_*Asx*_≤0.7 *hr* compared to larger *σ*_*Asx*_ Thus, details of the asexual development process affects gametocyte dynamics in an immune-state dependent way in the simulations. This issue is explored more in the Discussion below. Plots of analogous results for Non-CS model simulations are shown Additional file 1: Figure S11. The main difference from Figure 8 is that *PD*_*MG*_/*PD*_*Asx*_ has almost no dependence on *σ*_*Asx*_ if gametocytes are invisible to the model innate response.

### Discussion

The most important insight from the simulations studied in this report is that differing modalities of host immune response against gametocytes would affect their population dynamics in fundamentally different ways that may have implications for transmission. First, if there is no acquired immunity against gametocytes, the density of the asexual forms determines the density of the mature gametocytes, assuming that that after some triggering event, a set proportion of merozoites develop into sexual forms. The parasite-days *PD*_*MG*_ and maximum density *Max*_*MG*_ of mature gametocytes grow almost linearly with the asexual parasite days *PD*_*Asx*_ and maximum asexual density *Max*_*Asx*_, respectively. This is true even if the model innate response could remove gametocytes at the same clearance rate as the asexual forms, although in infections in which the host clears the parasite, *PD*_*MG*_ and *Max*_*MG*_ are reduced by the effects of the innate immunity. In simulated infections in which the host dies by anemia, the model innate response suppresses *PD*_*MG*_ and *Max*_*MG*_ by a factor of 10 to 100 in *P. vivax* infection, and a factor of 1000 in *P. falciparum* infections compared to the case of gametocytes being invisible to all immunity. However, for the model examined here, one should realized that death by severe anemia takes nearly three weeks at a minimum from primary release, and the model innate response is activated more on average than during infections in which the host clears the parasites. If innate responses are inhibited or exhausted, or if severe malaria is induced by a mechanism other than anemia with hyper-parasitemia, then the gametocyte levels in severe malaria might differ from the behavior suggested by the models. In addition, some researchers have suggested that the sequestration of immature gametocytes of *P. falciparum* (as discussed in the Background above) helps the immature forms evade host TNF pyrogenic response, thus moderating suppression of *P. falciparum* gametocytes by this mechanism of innate immunity [57].

For the immune modalities in which gametocytes are attacked by an antibody response, antibodies capable of clearing immature gametocytes would suppress the density of the transmissible mature forms due to “choking off” the source of future mature gametocytes. For the same value of the immune strength measure *IS*_*Gcy*_, such antibodies are much more effective at reducing overall numbers of the mature forms than antibodies directly against the mature forms. This was true for both *Plasmodium* species; in particular, *P. falciparum* is extremely vulnerable to having its potential transmission completely blocked by this immune modality in simulated infections due to the long time needed for its immature gametocytes to mature. Even when mature gametocytes are made, their density is suppressed much more than in *P. vivax* infections for the same values of *PD*_*Asx*_ and *IS*_*Gcy*_. Yet clinical measurements in malaria patients suggest that the densities of mature gametocytes of the two species are comparable [58], which leads to a question: how does *P. falciparum* produce enough mature forms to ensure transmission? One possibility is that the proportion of merozoites of this species that go down the sexual pathway is so large that despite enormous depletion of the immature forms, enough of them reach maturity at a level capable of causing transmission. A survey of blood from the bone marrow from a group of *P. falciparum* infected Gambian children found only ≈0.05*%* of erythrocytes in that compartment actually contained immature gametocytes, while ≈8*%* of the peripheral erythrocytes were infected with asexual forms [47], so a colossal rate of gametocytogenesis in *P. falciparum* infection seems unlikely. A second way that an antibody response could be mitigated is by sequestering of mature gametocytes in peripheral capillaries, thus boosting their density in the blood that a mosquito would uptake. Experimental evidence suggests that this happens in the rodent malaria caused by *P. chabaudi*[59]. Of course, some immature forms have to survive to maturity in order for this mechanism to be effective. The results above suggests a third way: perhaps the immature gametocytes of *P. falciparum* can evade host antibody responses by not showing surface antigens, or are vulnerable only for a fraction of their duration. A recent study found that immature gametocytes of *P. falciparum* do not form the “adhesive knob structures” typical seen on the surface of erythrocytes parasitize by the asexual forms of *P. falciparum* and express much less erythrocyte membrane protein PfEMP1 then the asexual forms [60]. As mentioned in the introduction, a study in Gambia found antibodies to antigens on the surface of erythrocytes parasitized with mature gametocytes of *P. falciparum*, but not to the immature gametocytes [20]. And there is some evidence that antigenic variation of a surface antigen may aid the survival of immature gametocytes [61], but more needs to be done to determine this.

A study on neurosyphilis patients treated with *P. falciparum* suggested that the probability that mosquitoes become infected after feeding on a patient is proportional to the gametocyte density [62], although the authors of that study noted that some patients with high gametocyte density failed to infect, while others with gametocyte density <10 *μL*^−1^ infected. Another study on malaria patients in Ghana did not indicate a clear correlation between gametocyte density and transmission to mosquitoes [63]. Furthermore, a study on neurosyphilis patients with *P. vivax* infection suggested that transmission success depends on only the density of male gametocytes [64]. These conflicting results suggest that while the probability that a patient can transmit the infection may be affected by *PD*_*MG*_ there must be other factors influencing transmission. The window of time when transmission is possible, *Dur*_*MG*_ may be just as important. The results reported above indicate that once mature gametocytes are produced, antibodies to the mature forms would be important in reducing *Dur*_*MG*_. Of course, such antibodies would still reduce *PD*_*MG*_, even if not to as large a degree as antibodies to the immature forms. Thus if a threshold density of mature gametocytes is needed to ensure transmission, or if mature gametocytes concentrate in subdermal capillaries, antibodies to the mature forms could still interfere with transmission.

Another insight from this study is that details of development of the asexual forms can combine with a host’s immune system dynamics and affect the production of mature gametocytes. Theoretical studies suggest that the presence of a quickly acting, quickly decaying innate immune response forces the synchronization of schizont rupturing [65], and synchronization of schizont rupture occurs in models examined here as well [30]. In addition, the circadian melatonin cycle of the host may also regulate the overall cycle of asexual development [66]. The synchronization of merozoite release is not directly set by *σ*_*Asx*_, the standard deviation in the population-averaged duration of the asexual intracellular stages, but *σ*_*Asx*_ sets the “sharpness” of this release. However, the value of *σ*_*Asx*_ in real infections is not clear. It was previously reported that the larger *σ*_*Asx*_ is, the larger the density of the asexual forms, and that this effect seems to be due to the presence of a quickly-acting innate response triggered by merzoite releases [30]. It was shown above that if the gametocytes are invisible to host immunity, *PD*_*MG*_/*PD*_*Asx*_ weakly decreases with increasing *σ*_*Asx*_ for a given *PD*_*Asx*_. However, if the model innate immunity could remove gametocytes, smaller *σ*_*Asx*_ tends to suppress *PD*_*MG*_/*PD*_*Asx*_, especially for *P. falciparum* infections with *PD*_*Asx*_ ≥ 300 *μL*^−1^*day* and *σ*_*Asx*_ ≤ 0.7 *hr*. If the rupturing of schizonts releases immune suppression factors [67], and if these factors help suppress damage to gametocytes, then the mass release of such factors when schizonts rupture in unison would preserve the efficiency of gametocyte production enough to ensure transmission even if *σ*_*Asx*_ ≤ 1 *hr*. Synchronization of schizont rupture and release of immune suppression factors during rupture may be processes evolutionarily shaped by intense host-parasite interactions.

## Conclusions

In order to elucidate the consequences of different host immune modalities against the gametocytes, this report consists of a theoretical population biological study of *Plasmodium* populations interacting with their human hosts. It is shown that antibodies against the immature gametocytes would suppress the density of mature gametocytes to a greater degree than antibodies that act directly against the mature forms for the same ability of antibody to clear their targets. This would be true for both *P. vivax* and *P. falciparum* infections; in particular, *P. falciparum* is very vulnerable to having its potential transmission completely blocked by this immune modality. Antibodies directly against mature forms would still interfere with transmission by reducing the window of transmissibility during and after a bout of malaria. In addition to the direct effects of immune modalities upon gametocytes, it was also found that the standard deviation in the development time of individual asexual intraerythrocytic parasites can affect the efficiency at which tranmissible gametocytes are produced due to the interplay between the development of the asexual forms and host innate immunity.

## Methods

### Basic algorithm for modeling dynamics of aging populations

Individuals in a population age with some average duration *D* before progressing to the next stage of life development or senescence, and not all individuals necessarily take the same amount of time to age. The youngest members of the population might be created at a time-varying rate *s*. In population modeling, this is a time delay system which can be difficult to solve [68]. A useful formalism for approximating pathogen population dynamics was adapted by Lloyd from ecology [69,70]. The idea is to introduce a set of coupled ordinary different equations (ODEs) that govern the time evolution of a set of of fictitious variables, *P*_1_,*P*_2_,*P*_3_,…*P*_*N*_. The ODEs are chosen so that the rate at which individuals leave the population for the next stage (or death) is approximately a normal function of time *t* with mean *D* and variance *σ*^2^=*D*^2^*N*^−1^. Ignoring environmental influences which might remove the population, the ODEs are

(1) $$\begin{array}{l} P_{1}^{\prime }=s(t)-\Lambda P_{1}\;(\text{where}\;\Lambda =ND^{-1})\\ P_{n}^{\prime }=\Lambda (P_{n-1}-P_{n}),\;1<n\leq \mathrm{N.} \end{array}$$

In this formalism, the total population is given by the sum of all *P*_*n*_, and *s* is the rate (source term) at which new individuals enter the population. The tangible quantities *D* and *σ* set *N*, the total number of components. (If *D*=*σ*, then the system of Equations 1 reduces to a single equation describing exponential decay.) As mentioned above, this formalism has been used to describe within-host populations of the asexual *Plasmodium* cells interacting with various systems of the host [29-31]. For this report, this formalism has been adapted to include gametocytes as well. The populations considered in this report are shown schematically in Figure 1, along with the corresponding values of *D* and *σ*. (All population sizes are stated as number per *μL* of blood.) Because the full system of differential equations for the modal formalism involves many hundreds of components, it is given in detail in Additional file 2. A qualitative description is given below for each of the major parts of the model.

### Model for asexual parasite population dynamics

As shown in Figure 1, five populations of asexual parasite cells which are morphologically distinct from each other were considered: (1) ring stage, (2) early trophozoites, (3) late trophozoites, (4) schizonts, and (5) merozoites. The dynamics of each of these populations is described by a set of equations similar to Eq. 1 with the addition of terms due to host immune responses described below. (See equation 6 in Additional file 2). The duration and variance of each of the intracellular stages (1-4) were taken to be the same, with duration *D*_*Asx*_ = 12 hr, but with the variance $\sigma_{\text{Asx}}^{2}$ varied from simulation to simulation. (The sampling of parameters varied from simulation to simulation as explained and tabulated in Subsection “Sampling the parameter space” below). Since merozoites are short lived in blood, with duration *D*_*μ*_=0.1 *hr*[71], just one compartment was used for them, *μ*, and take *σ*_*μ*_=*D*_*μ*_. There are two sources for the asexual population: (1) the primary release of merozoites from the liver, thus triggering the blood infection phase of the disease, the (2) merozoite that are released from the bursting schizonts, with *p* number of merozoites released on average from each schizont. The simulated infection begins with the primary release of merozoites, an event that takes just a few hours to complete [72]. For every simulated infection, it was assumed that the rate of primary release was 0.002 (*μLhr*)^−1^, maintained for the one hour. This is equivalent to 10^4^ merozoites being released into an adult human with blood volume 5×10^6^*μL*. Whether from primary release or from bursting schizonts, the rate of creation for new trophozoites is *μζV*, where *μ* is the density of merozoites, *ζ* is the binding affinity of merozoites to their target erythrocyte, and *V* is the density of the targeted erythroyctes (reticulocytes only for *P. vivax*, all red blood cells for *P. falciparam*). (Remember that *μ* is also time dependent.) For this report, asexual density *Asx* means the sum of the ring stage, early and late trophozoite, and schizont blood densities. (Although the later stages of the intracellular asexual forms sequester in *P. falciparum* infection, *Asx* was taken as a measure of the full load of the asexual forms on the host.) The parasite days for the asexuals mentioned in the discussion, *PD*_*Asx*_, would be the average of *Asx* during infection in host times the duration of infection in host. If *Asx* ≥ 0.01 *μL*^−1^, then it is assumed that a fraction *r*=0.05 of new infected erythrocytes are committed to the cryptic sexual pathway.

Experimental and clinical data put strong constraints on the values of *p* and *ζ*. Define the initial reproduction rate *R*_0_ as the average number of progeny an individual parasite would have after primary release of merozoites and before host immune responses. One can show that *R*_0_ to *p*, *ζ*, and *D*_*μ*_ are closely related [29]:

(2) $$\zeta \;V_{0}\;D_{\mu }=R_{0}\;(p-R_{0})$$

(Here *V*_0_ is the initial density of vulnerable red blood cells.) Observations of parasite growth in neurosyphilis patients treated with malaria therapy [64,73] as well as in inoculated volunteers [74] indicate that *R*_0_≈15 for both *P. vivax* and *P. falciparum*. Photographs of bursting *P. falciparum* schizonts indicate *p* ≥ 16 [75]. Thus, for this report *p* = 16, *R*_0_=15. Equation 2 fixes *ζ* to 3×10^−5^*μLhr*^−1^ for *P. falciparum* and 2.4×10^−3^*μlhr*^−1^ for *P. vivax*.

It is assumed that the four intracellular stages are attacked by the innate immune response of the host, with time-dependent clearance rate *χ*_*Inn*_. Host acquired responses in malaria are complicated, (see [76] and [77] for review), but for simplicity, it is assumed for this report that the main antibody response is against the schizont stage with time-dependent clearance rate *χ*_*Sch*,*Ab*_. (Immune dynamics models are explained in more detail in Subsection “Model for immune response dynamics” below.) Also for simplicity, the variation in surface antigens which *P. falciparum* employs for immune evasion was not considered [78].

### Model for population dynamics of sexual forms

As mentioned above, two very different models of gametocytogenesis were considered, the “cryptic sexual” (CS) model and the “non-cryptic sexual” (non-CS) model. As illustrated in Figure 1, in the CS model there is a set of parasite stages that morphologically resemble the asexual stages, but are committed to eventual development into overt gametocytes. The ring stage, early trophozoites, late trophozoites, schizonts, and merozoites each have cryptic sexual counterparts. (See equation 7 in Additional file 2.) An erythrocyte invaded by a cryptic sexual merozoite becomes a gametocyte. For each simulation, it was assumed that the values corresponding to *D*_*Asx*_, *σ*_*Asx*_, *N*_*Asx*_, *D*_*μ*_, *p*, and *ζ* are the same as for the asexual populations. Based on studies of gametocyte duration [50,51]. the immature gametocyte duration *D*_*IG*_ was set to 216hr for *P. falciparum*, with *σ*_*IG*_ = 24 hr. For *P. vivax*, *D*_*IG*_ was set to 72 hr with *σ*_*IG*_ = 12 hr. For simplicity, the mature gametocyte population was represented with a single compartment, MG with exponential decay, *D*_*MG*_ = *σ*_*MG*_ = 156hr, based on a study of gametocyte dynamics in malaria therapy patients [56]. (The actual point at which a cryptic sexual form emerges is not yet clear, but the CS model is a extreme formulation of the cryptic sexual thesis.) It was assumed that the cryptic sexual forms are subject to the same innate clearance rate *χ*_*Inn*_ and antibody clearance rate *χ*_*Sc*,*Ab*_. In the Non-CS model, there are no cryptic sexual stages at all; a certain proportion (*r*=0.05) of erythrocytes invaded by regular merozoites directly become gametocytes once *Asx* ≥ 0.01 *μL*^−1^ (See equation 8 in Additional file 2.)

Four immune modalities were considered for gametocytes in this report for both *P. falciparum* and *P. vivax*, and for both the CS and Non-CS models: (1) no host immune responses to gametocytes at all, (2) no antibody response to gametocytes, but an innate response with the same clearance rate *χ*_*Inn*_ as for the asexuals, (3) an innate response to all gametocytes with clearance rate *χ*_*Inn*_, and an antibody response to the immature gametocytes but not to mature gametocytes (and different from the one to schizonts) with clearance rate *χ*_*IG*,*Ab*_, and (4) an innate response to all gametocytes with clearance rate *χ*_*Inn*_, and an antibody response on the mature but not immature gametocytes, with clearance rate *χ*_*MG*,*Ab*_. The parameters for antibody responses to gametocytes are chosen independently to the response against schizonts. See “Model for immune response dynamics” below.

### Model for red blood cell dynamics

Three populations were used to describe the red blood cells: (1) reticulocytes, the youngest of the erythrocytes, (2) mature red blood cells, and (3) senescent red blood cells ready to be removed by phagocytosis in the spleen, liver or bone marrow [79]. (See equation 9 in Additional file 2.) Based on hematological studies [80], the respective durations of the stages are taken as *D*_*Re*_ =36 *hr* (with *σ*_*Re*_ =6 *hr*),), *D*_*Ma*_ =2796 *hr* (with *σ*_*Ma*_ =148 *hr*), and *D*_*Se*_ =48 *hr* (with *σ*_*Se*_ =12 *hr*). As mentioned above, if *V* is the density of the erythrocyte stage or stages vulnerable to the merozoite invasion, then the total number of erythrocytes loss to infection by merozoites is *ζμV*. The basal erythrocyte density for a healthy adult was assumed to be 5×10^6^*μL*^−1^, and if the total density of uninfected erythrocytes drop to under 60% of this value, it was assumed that the host died.

The rate of production of new reticulocytes from the bone marrow, E, is the source term for the erythrocytes and has its own dynamics. (See equation 10 in Additional file 2). In a healthy human, the erythropoietic system makes new cells at a basal rate $E_{0}$ to maintain the red blood cell density at 5×10^6^*μ*L ^−1^, (≈1736 (*μ*L *hr*)^−1^). In the event of blood loss, the erythropoietic system can boost the rate of RBC production up to $\approx 5\times E_{0}$ within a couple of days [80]. However, both *P. vivax* and *P. falciparum* can modulate erythropoiesis, causing a dysfunction of erythropoiesis [28] probably through several mechanisms [81-84]. To account for these effects, erythropoietic source dynamics of the model were designed to be controlled by feedback from the dynamics of the RBC populations, so if there were no dyserythropoeisis, the rate of increase E itself is proportional to *λ*_*ES*_×*ζμV*. Here $\lambda_{\text{ES}}^{-1}=48\;\text{hours}$. To account for dyserythropoiesis, an offset *δ*_*Dys*_*ζμV* was subtracted from the growth rate in E. For simplicity, it was assumed that the factor *δ*_*Dys*_ had no dynamics of its own although it varied form from simulation to simulation; see Subsection “Sampling the parameter space” below. A value *δ*_*Dys*_=0 means there is no dyserythropoiesis.

### Model for immune response dynamics

As indicated in Figure 1, host immune responses were modeled in a very phenomenological manner: the presence of some stage of the parasite triggers the actuator stage, which is self-amplifying and eventually triggers the attacker component that removes some stage of the parasite. The entire response becomes self-limiting. The equation of dynamics are modifications of Eq. 1 above which incorporate the self-amplification and self-limiting. Although a highly simplified model of real immune processes, it does capture real aspects of human immune reactions in malaria [30], and is similar to models used to describe immune responses against viruses [85]. Up to three immune responses were considered for each simulation: (1) a quickly acting, quickly decaying innate response, (2) a slow-to-start but long lasting antibody response to intracellular asexual parasites, (3) a slow-to-start but long lasting antibody response to gametocytes. (As discussed below, in some simulations gametocytes were invisible to the host immune systems.) Consider the innate response first: the actuator $A_{\text{Inn}}$ is present at some background level $A_{\text{Inn},0}$ when the response is quiet. Various studies indicate that malarias can trigger a rapid and strong cytokine response in the host upon bursting of schizonts, which then decays on a time scale of ≈1 *hr* until triggered again [22,86-88]. Thus, in models reported here the presence of merozoites triggers the production of more actuator, which in turn triggers the attacker component that clears its targets at rate *χ*_*Inn*_. A buildup of $A_{\text{Inn}}$ or *χ*_*Inn*_ then limits the response. The limit on growth of the actuator is set by parameter $\Delta A_{\text{Inn},\text{Mx}}=10\;\mu l^{-1}$. The limit on growth of the attacker is set by maximum clearance rate *χ*_*Inn*,*Mx*_, which was varied from simulation to simulation. The level of merozoites that triggers the model innate response was varied from simulation to simulation; see Subsection “Sampling the parameter space” below. The values of *D* and *σ* used for the actuator and attacker components, as well as the self-amplification and self-limiting terms were chosen to emulate the dynamics seen in the cytokine responses reported in malaria patients [87,88]. (See equation 11 in Additional file 2.)

The actuator-attack formalism was adapted to model host antibody responses as well, since antibody responses also have feedback [89]. Acquired immunity responses take some days to activate once the triggering antigen is sensed, so a delay stage was included in the antibody models with its own *D* and *σ*. The values of the *D* and *σ* used for actuator and delay stages (as shown in Figure 1) as well as the self-amplification and self-limiting terms were chosen to emulate the T-cell dynamics seen acquired immunity responses [85]. (See equation 12 in Additional file 2.) Every simulation incorporated an antibody response to the schizont stage of the asexuals. As discussed above, some simulations incorporated an additional antibody response to the gametocytes, either against the immature forms or the mature forms. For each antibody response, the density of the targeted parasite stage, and the maximum clearance rate of the response were parameters varied from simulation to simulation. In addition, studies indicate that antibodies to malaria antigens can last from a couple of months to years [90,91], so *D* for the attacker stage of the antibodies was varied from simulation to simulation as well; see Subsection“Sampling the parameter space” below. For simplicity, the effects of memory B cells were not considered.

### Sampling the parameter space

Several model parameters values were varied from simulation to simulation, either because the parameters vary strongly from patient to patient, or else their values are not known. For a full listing of the parameters that were varied from simulation to simulation, the range of values used, and the equations in which they appear, see Table S1 in Additional file 2. For a given class of sexual pathway, species, and immune structure, the Latin hypercube algorithm [92] was used to sample among the relevant parameters in the following manner: let $p_{1},p_{2},\ldots p_{M}$ be the parameters varied for given model class. The plausible range for each *P*_*n*_ is divided into ten equal intervals. A 10×*M* matrix M was defined in which the columns consist of a random ordering of the integers 1 through 10, with no integer repeated in a column. The integers are associated with the parameter intervals as follows: integer *k*=*M*_*i*,*n*_ labels interval *k* for parameter *P*_*n*_. The first simulation uses values of $p_{1},p_{2},\ldots p_{M}$ chosen randomly within the intervals labeled by $M_{1,1},M_{1,2},\ldots M_{1,M}$. The second simulation uses values chosen randomly within the intervals labeled by $M_{2,1},M_{2,2},\ldots M_{2,M}$. This procedure is repeated until values in the intervals labeled by $M_{10,1},M_{10,2},\ldots M_{10,M}$ are used. The order of the integers in each column is scrambled to repeat the procedure again. Thus, for a given class of sexual pathway, species, and immune structure, 2000 randomized versions of M were used so that there would be 20000 simulations altogether. The Latin hypercube algorithm was used to attempt uniform sampling of the parameter space for a class of models, although with so many variable parameters the sampling could not be perfect.

### Solving the model equations

To solve the ODE system, a fifth-order Runge-Kutta-Fehlberg algorithm with adaptive step-size control was used for time integration [93,94]so that the difference between the fourth- and fifth-order solutions for each component of the ODE systems was less than one part in 10^6^. Since cells are discrete entities the following constraint was enforced: if any density of a cell population indicates less than one cell in a normal blood volume of 5×10^6^*μl*, then all components associated with that population are set to zero. See Additional file 2 for more details on how this was enforced. A simulation stopped when (1) the host died, (2) all parasite stages are cleared, (3) simulated time reached 3 years, or (4), the adaptive time stepping algorithm itself could not converge with the desired precision. With the choice of parameters used, only for *P. vivax* CS models did (4) occur, affecting 39 out of 8×10^4^ simulations.

### Ethical clearance

The study involved no experimental research on humans or animals.

## Competing interests

The authors declare that they have no competing interests.

## Authors’ contributions

PGM and FEM conceived of the study, and KCW suggested adding the cryptic sexual model of gametocytogenesis. All three authors helped in the detailed design of the theoretical population biological models. PGM wrote the computer codes which implemented the theoretical models and collated the results of the simulations. All three authors participated in writing the manuscript and approving its final form. All authors read and approved the final manuscript.

## Supplementary Material

Additional file 1 **First supplemental file to the report “Potential host immune constraints upon malaria transmission: insights from population biology of the within-host parasites”.** This PDF file contains the supplemental figures that have to do with the dynamics of the asexual populations and with the Non-CS model of gametocytogenesis. Click here for file

Additional file 2 **Second supplemental file to the report “Potential host immune constraints upon malaria transmission: insights from population biology of the within-host parasites”.** This PDF file contains three discussions: (1) a brief introduction to matrix and vector notation for those unfamiliar with such notation (which helps to state the model formalism concisely), (2) the full set of equations for the model formalism, and (3) more justification as to why antibodies to immature gametocytes would be more efficient in suppressing the density of transmissible gametocytes than antibodies directly against the transmissible forms. Click here for file
