# Supplementary material for: Host immune constraints on malaria transmission: insights from population biology of within-host parasites
Source: Malar J. 2013 Jun 15;12:206. doi: 10.1186/1475-2875-12-206 (PMC3691866; doi:10.1186/1475-2875-12-206)
Supplement: Additional file 1 — First supplemental file to the report “Potential host immune constraints upon malaria transmission: insights from population biology of the within-host parasites”. This PDF file contains the supplemental figures that have to do with the dynamics of the asexual populations and with the Non-CS model of gametocytogenesis. [file 1475-2875-12-206-S1.pdf]

First supplemental file to the report "Potential host immune constraints upon malaria transmission: insights from population biology of the within-host parasites"

Philip G. McQueen[1,*], Kim C. Williamson[2,3] F. Ellis McKenzie[4],
1 Mathematical and Statistical Computing Laboratory, Division of Computational Bioscience, Center for Information Technology, National Institutes of Health, Bethesda, Maryland, USA
2 Loyola University of Chicago, Department of Biology, Chicago, Illinois USA
3 Laboratory of Malaria and Vector Research, National Institute of Allergy and Infectious Diseases, National Institutes of Health, Bethesda, Maryland, USA
4 Fogarty International Center, National Institutes of Health, Bethesda, Maryland, USA
∗ E-mail: mcqueenp@mail.nih.gov

Abstract

This file contains the supplemental figures to the report "Potential host immune constraints upon malaria transmission: insights from population biology of the within-host parasites"

A Note on Measuring the Strength of the Immune Response to Asexual Populations

For Figures S1, S3, S4 and S5 below, we use as a measure of the strength of total model host immune responses to the asexual forms the quantity IS_{Asx}:

$$IS_{Asx} = \sqrt{(\frac{\chi_{Inn,Mx}}{\mu_{Th}})^2 + (\frac{\chi_{Ab,Sc,Mx}}{Sc_{Th}})^2}$$

Here μ_{Th} is the merozoite density that triggers the model innate response of the host, and $\chi_{Inn,Mx}$ is the maximum rate of clearance by that response. Similarly, Sc_{Th} is the density of the schizonts that triggers the model antibody response against the response, and $\chi_{Ab,Sc,Mx}$ is the maximum clearance rate of that response. See Methods section of main text, and equations 11 and 12 in Additional File 2.

As can be seen in Figures S1, S2, S3, and S4, the properties of the asexual populations are invariant to the immune modalities against the gametocytes. This is to be expected, since there is no feedback from the gametocyte population back to the asexual populations in either the cryptic sexual ("CS") or Non-cryptic sexual ("Non-CS") models once gametocytogenesis is triggered.

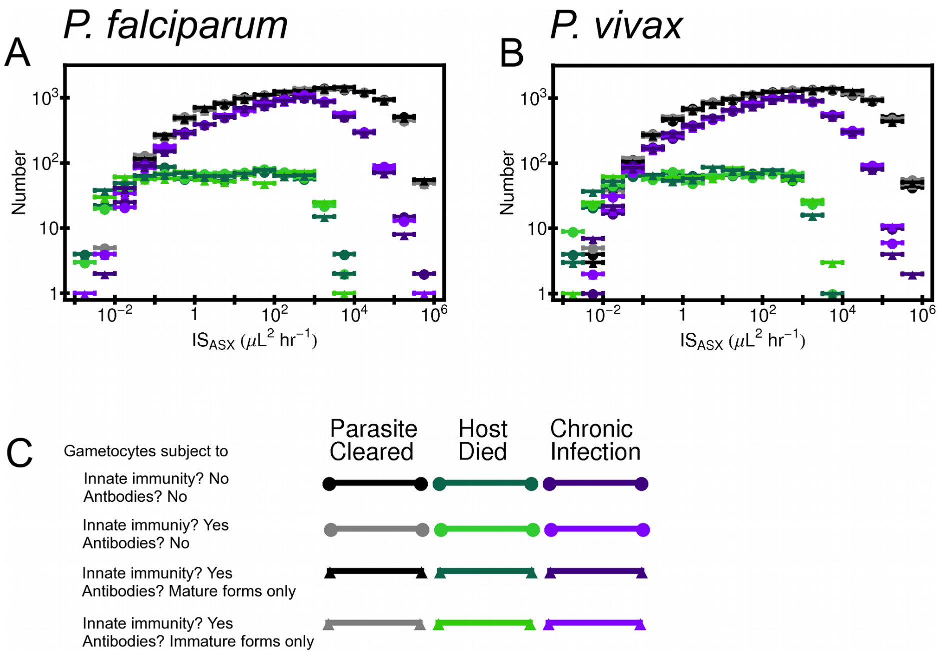

Figure S1: Number of Simulations as a Function of Immune Strength Measure IS_{Asx}, Cryptic Sexual (CS) Model. Curves for each of four immune modalities on the gametocytes is shown on each plot. Simulations are binned by the logarithm of IS_{Asx}. The horizontal bars show the width of each bin. (A) Number of simulated *P. falciparum* infections in each IS_{Asx} bin. (B) Number of simulated *P. vivax* infections in each IS_{Asx} bin. (C) Color code for immune modality on gametocytes and outcome of simulated infections. Abbreviations: "Parasite Cleared": host cleared all parasite forms within three years after primary release of merozoites from the liver. "Host Died": host died of anemia within three years of primary release. "Chronic Infection": host alive but infection not cleared by three years after primary release.

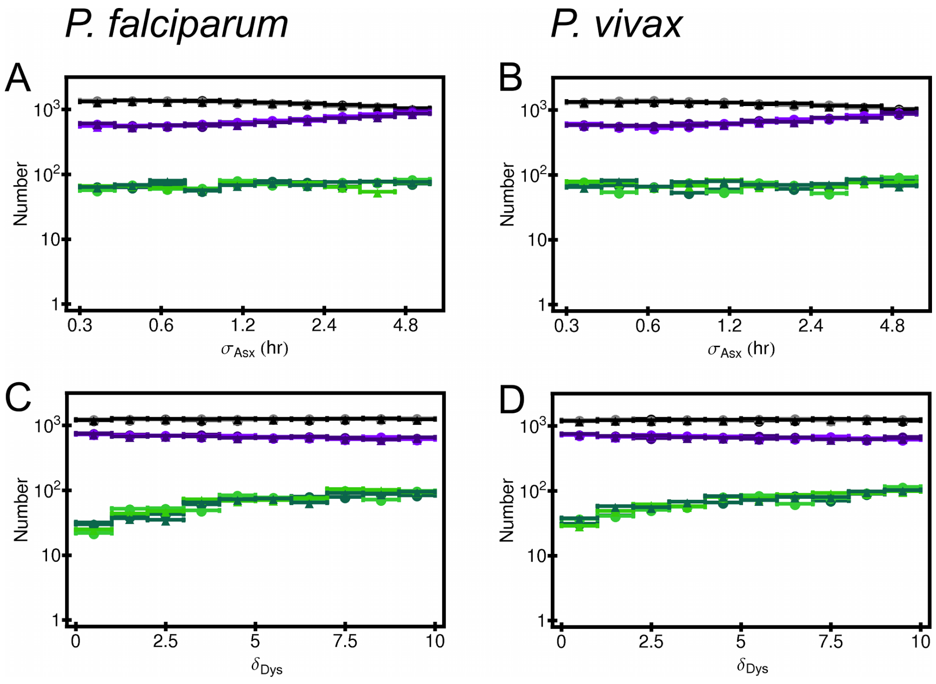

Figure S2: Number of Simulations as a Function of Certain Model Parameters, CS Model. For panels (A) and (B), simulations are binned by the logarithm of σ_{Asx}, the standard deviation in the duration of the intracellular asexual stages. For panels (C) and (D), simulations are binned by the values of the dyserythropoiesis δ_{Dys}. (See "Model for red blood cell dynamics" in Methods section of main text.) The horizontal bars show the width of each bin. Curves for each of four immune modalities on the gametocytes is shown on each plot. (A) Number of simulated *P. falciparum* infections in each σ_{Asx} bin. (B) Same as (A), except for simulated *P. vivax* infections. (C) Number of simulated *P. falciparum* infections in each δ_{Dys} bin. (D) Same as (A), except for simulated *P. vivax* infections. Color code for immune modality on gametocytes and outcome of simulated infections is the same as for Figure S1.

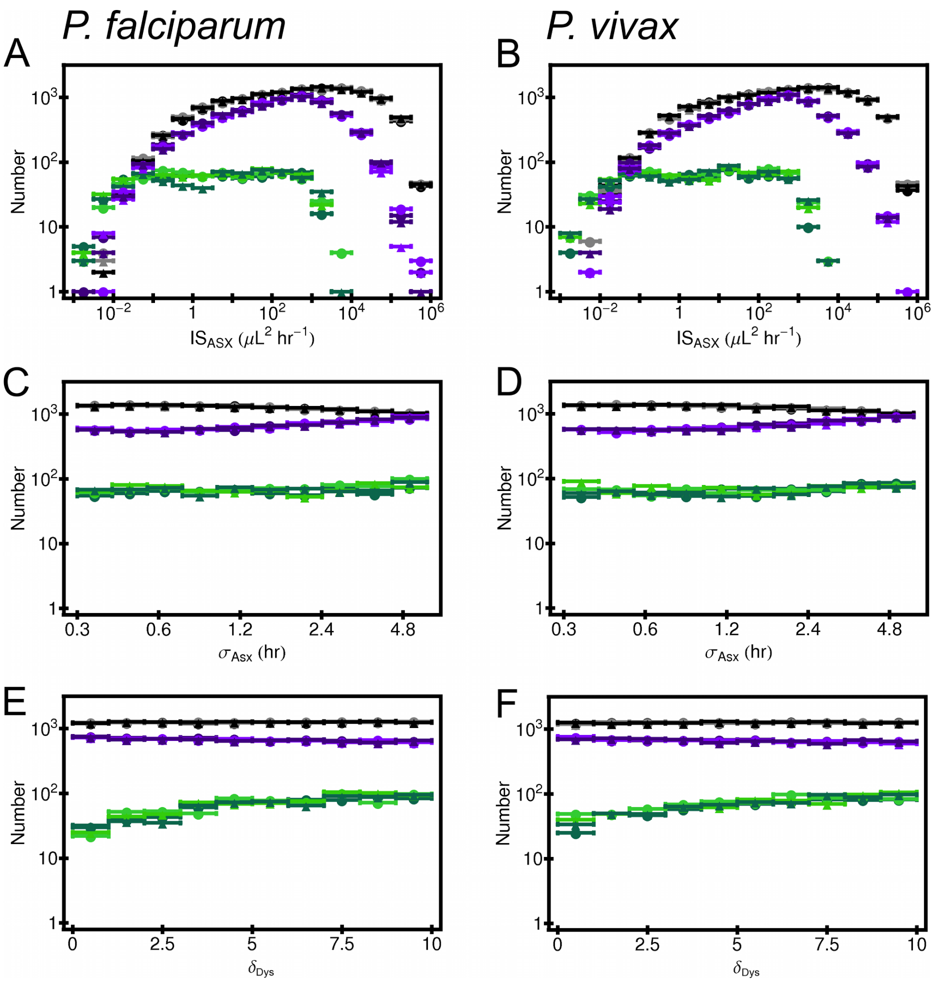

Figure S3: Number of Simulations as a Function of Various Model Parameters, Non-Cryptic Sexual (Non-CS) Model. The plots shown in this figure were constructed the same as the plots in Figure S1 and S2, except that these are for Non-CS model simulations. Curves for each of four immune modalities on the gametocytes is shown on each plot. (A) Number of simulated *P. falciparum* infections in each IS_{Asx} bin. (B) Same as (A), except for simulated *P. vivax* infections. (C) Number of simulated *P. falciparum* infections in each σ_{Asx} bin. (D) Same as (C), except for simulated *P. vivax* infections. (E) Number of simulated *P. falciparum* infections in each δ_{Dys} bin. (F) Same as (E), except for simulated *P. vivax* infections. Color code for immune modality on gametocytes and outcome of simulated infections is the same as for Figure S1.

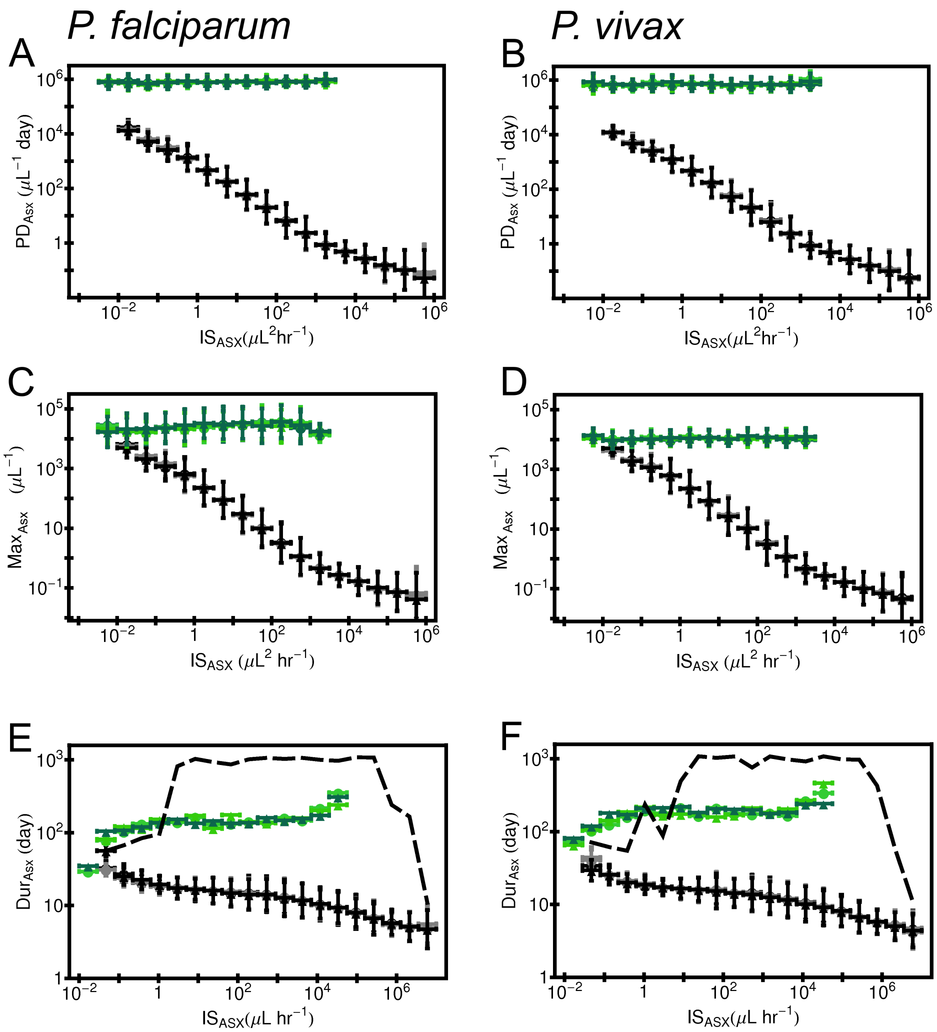

Figure S4: Density of Intracellular Asexual Parasites versus Measure of Host Immune Strength IS_{Asx}, CS Models
The simulations were binned by the logarithm of the measure of host immune strength against asexual forms, IS_{Asx}. The horizontal bars show the bin width. Curves for each of four immune modalities on the gametocytes is shown on each plot. (A) Logarithmic average of parasite-days of intracellular asexual forms PD_{Asx} versus IS_{Asx} for simulated *P. falciparum* infections that are resolved within three years of simulated time. (B) Same as (A), except for simulated *P. vivax* infection. (C) Logarithmic average of maximum blood density during infection of intracellular asexual forms Max_{Asx} versus IS_{Asx} for simulated *P. falciparum* infections that are resolved within three years of simulated time. (D) Same as (C), except for simulated *P. vivax* infection. (E) Logarithmic average of duration of intracellular asexual forms Dur_{Asx} versus IS_{Asx} for simulated *P. falciparum* infections that are resolved within three years of simulated time. (F) Same as (E), except for simulated *P. vivax* infection. Color code for immune modality on gametocytes and outcome of simulated infections is the same as for Figure S1, except that results from chronic infections are not plotted. In (E) and (F), the dashed line is the maximum Dur_{Asx} found for any infection (in each IS_{Asx} bin) for which the host clears the parasite.

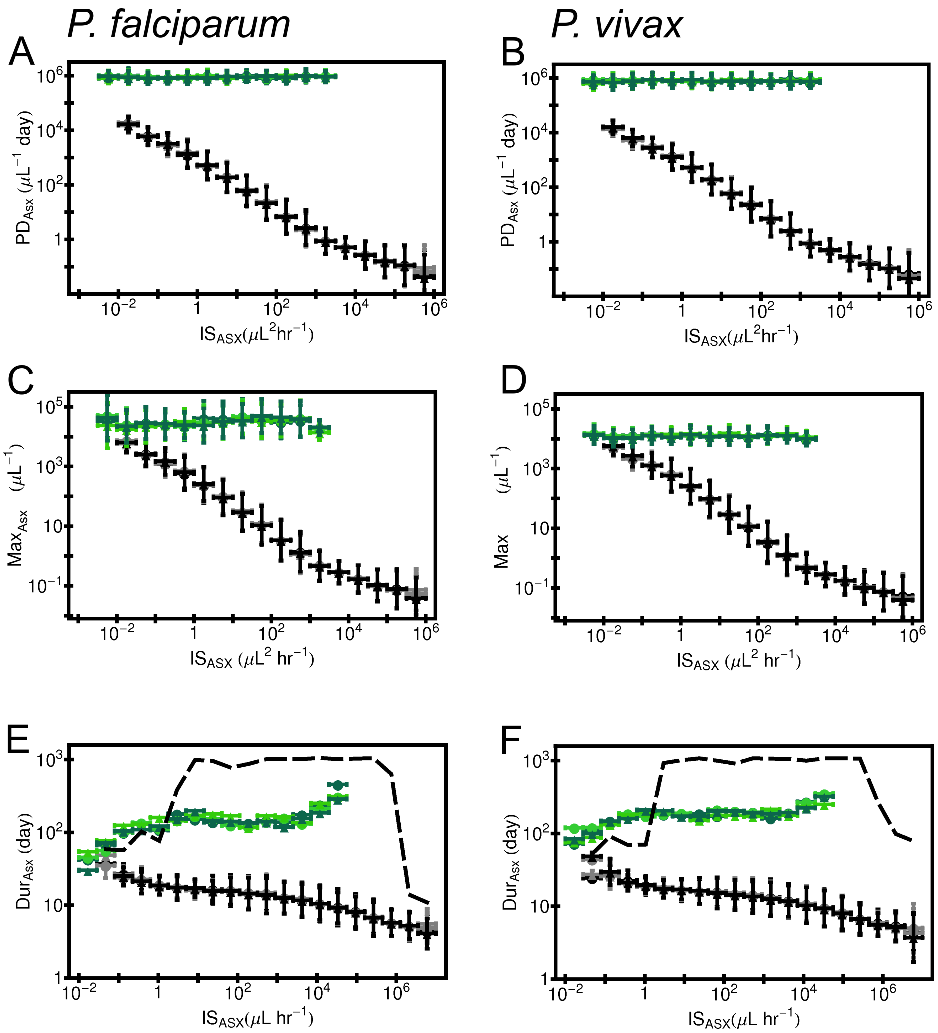

Figure S5: Density of Intracellular Asexual Parasites versus Measure of Host Immune Strength IS_{Asx}, Non-CS Models

The simulations were binned by the logarithm of the measure of host immune strength against asexual forms, IS_{Asx}. The horizontal bars show the bin width. Curves for each of four immune modalities on the gametocytes is shown on each plot. (A) Logarithmic average of parasite-days of intracellular asexual forms PD_{Asx} versus IS_{Asx} for simulated *P. falciparum* infections that are resolved within three years of simulated time. (B) Same as (A), except for simulated *P. vivax* infection. (C) Logarithmic average of maximum blood density during infection of intracellular asexual forms Max_{Asx} versus IS_{Asx} for simulated *P. falciparum* infections that are resolved within three years of simulated time. (D) Same as (C), except for simulated *P. vivax* infection. Color code for immune modality on gametocytes and outcome of simulated infections is the same as for Figure S1, except that results from chronic infections are not plotted. (E) Logarithmic average of duration of intracellular asexual forms Dur_{Asx} versus IS_{Asx} for simulated *P. falciparum* infections that are resolved within three years of simulated time. (F) Same as (E), except for simulated *P. vivax* infection. Color code for immune modality on gametocytes and outcome of simulated infections is the same as for Figure S1, except that results from chronic infections are not plotted. In (E) and (F), the dashed line is the maximum Dur_{Asx} found for any infection (in each IS_{Asx} bin) for which the host clears the parasite.

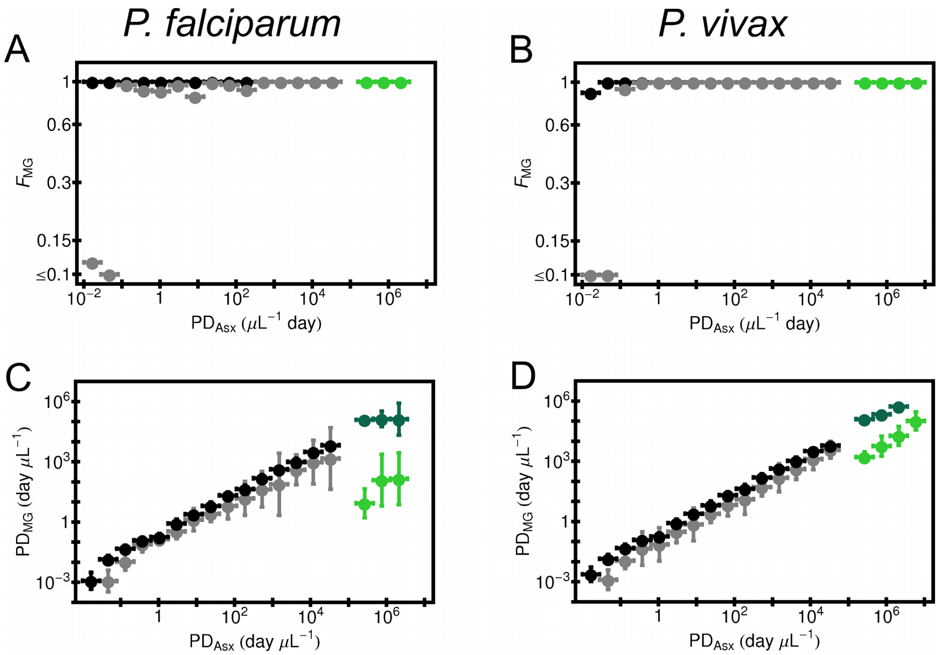

Figure S6: Efficiency of mature gametocytes production versus host exposure to asexual forms if gametocytes unaffected by acquired immunity, Non-CS Models Both immature and mature gametocytes are unaffected to the host acquired immunity. The simulations were binned by the logarithms of the parasite-days of intracellular asexual forms, PD_{Asx}; see text for definition. The horizontal bars show the bin width. (A) Fraction of simulations making mature gametocytes F_{MG} versus PD_{Asx} for simulated *P. falciparum* infections. (B) Same as (A), except for simulated *P. vivax* infections. (C) Logarithmic average of mature gametocyte parasite-days PD_{MG} versus PD_{Asx} for those simulated *P. falciparum* infections in which mature gametocytes are produced. Vertical bars are the standard deviation in the logarithmic average. (D) Same as (C) except for simulated *P. vivax* infections. Color code for immune modality on gametocytes and outcome of simulated infections are from the set shown in Figure S1. Note: for each of two immune classes and host fates color coded, results are shown only for bins which contained 10 or more simulations.

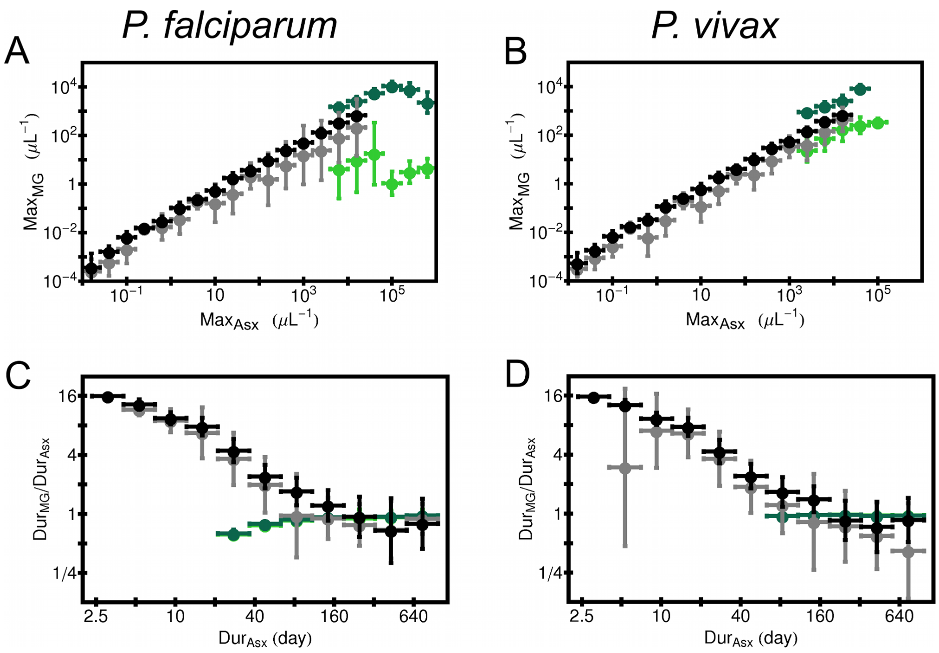

Figure S7: Some clinically observable properties of mature gametocytes if gametocytes unaffected by acquired immunity, Non-CS Models Immature and mature gametocytes unaffected by host acquired immunity response. (A) Plot of maximum blood density of mature gametocytes, Max_{MG}, versus maximum density of intracellular asexual forms, Max_{Asx} for simulated *P. falciparum* infections. The simulations were binned by the logarithm of Max_{Asx}, and horizontal bars show the width of each bin. The logarithmic average of Max_{MG} are computed for each bin for each of the four types of models color coded. Only simulations that produced mature gametocytes are included, and results are only shown for bins that contained 10 or more simulations. (B) Same as (A), except for simulated *P. vivax* infections. (C) Plot of ratio of duration of mature gametocytes in the host Dur_{MG} to duration of asexual forms Dur_{Asx} versus Dur_{Asx} for simulated *P. falciparum* infections. The simulations were binned by the logarithm of Dur_{Asx}, and horizontal bars show the width of each bin. The logarithmic average of Dur_{MG}/Dur_{Asx} are computed for each bin for each of the four types of models color coded. Only simulations that produced mature gametocytes are included, and results are only shown for bins that contained 10 or more simulations. (D) Same as (C), except for simulated *P. vivax* infections Color code for immune modality on gametocytes and outcome of simulated infections are from the set shown in Figure S1.

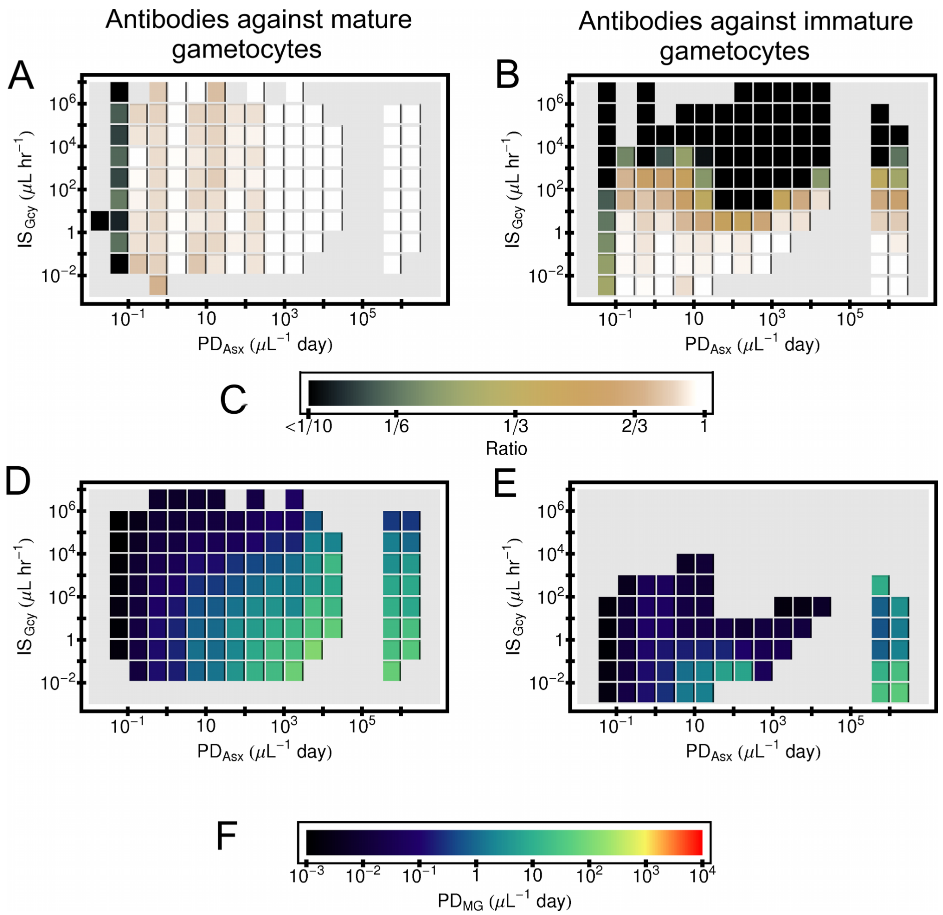

Figure S8: Effects of gametocyte-specific antibodies in simulated *P. falciparum* infection on gametocyte levels, Non-CS Models Results from simulated *P. falciparum* infections that resolved within three years from primary release of merozoites were binned by parasite-days of the intracellular asexual forms, PD_{Asx}, and also by a measure of the ability of the antibody response to clear their target, IS_{Gcy}. See text for definitions. The horizontal length of an individual block shows the bin size in PD_{Asx}, and the vertical extent of a block show the bin size in IS_{Gcy}. Results are only shown for bins that contained 10 or more simulations. (A) Fraction of simulations making mature gametocytes if a model antibody response attacks mature gametocytes. (B) Same as (A), except a model antibody response attacks immature gametocytes. (C) Color code for panels (A) and (B). (D) Logarithm mean of parasite days of mature gametocytes PD_{MG} for simulated infections producing mature gametocytes if a model antibody response attacks mature gametocytes. (E) Same of (D) except a model antibody response attacks immature gametocytes. (F) Color code for panels (D) and (E). Note: host died in all simulate infections for which $PD_{Asx} > 10^5 \mu L^{-1} day$, and parasite was cleared in all simulated infections if $PD_{Asx} < 10^5 \mu L^{-1} day$

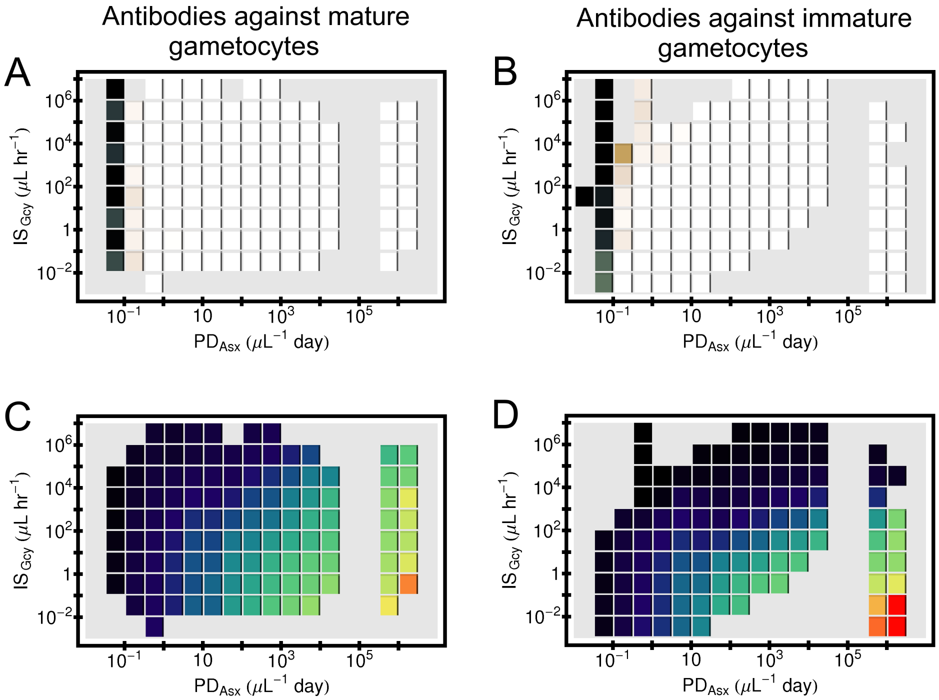

Figure S9: Effects of gametocyte-specific antibodies in simulated *P. vivax* infection on gametocyte levels, Non-CS Models

Results from simulated *P. vivax* infections that resolved within three years from primary release of merozoites were binned by parasite-days of the intracellular asexual forms, PD_{Asx}, and also by a measure of the ability of the antibody response to clear their target, IS_{Gcy}. See text for definitions. The horizontal length of an individual block shows the bin size in PD_{Asx}, and the vertical extent of a block show the bin size in IS_{Gcy}. Results are only shown for bins that contained 10 or more simulations. (A) Fraction of simulations making mature gametocytes if a model antibody response attacks mature gametocytes. (B) Same as (A), except a model antibody response attacks immature gametocytes. Color code for panels (A) and (B) is the same as for panels (A) and (B) in Figure S8. (C) Logarithm mean of parasite days of mature gametocytes PD_{MG} for simulated infections producing mature gametocytes if a model antibody response attacks mature gametocytes. (D) Same of (C) except a model antibody response attacks immature gametocytes. Color code for panels (C) and (D) are the same as for panels (D) and (E) in Figure S8. Note: host died in all simulate infections for which $PD_{Asx} > 10^5 \mu L^{-1} day$, and parasite was cleared in all simulated infections if $PD_{Asx} < 10^5 \mu L^{-1} day$

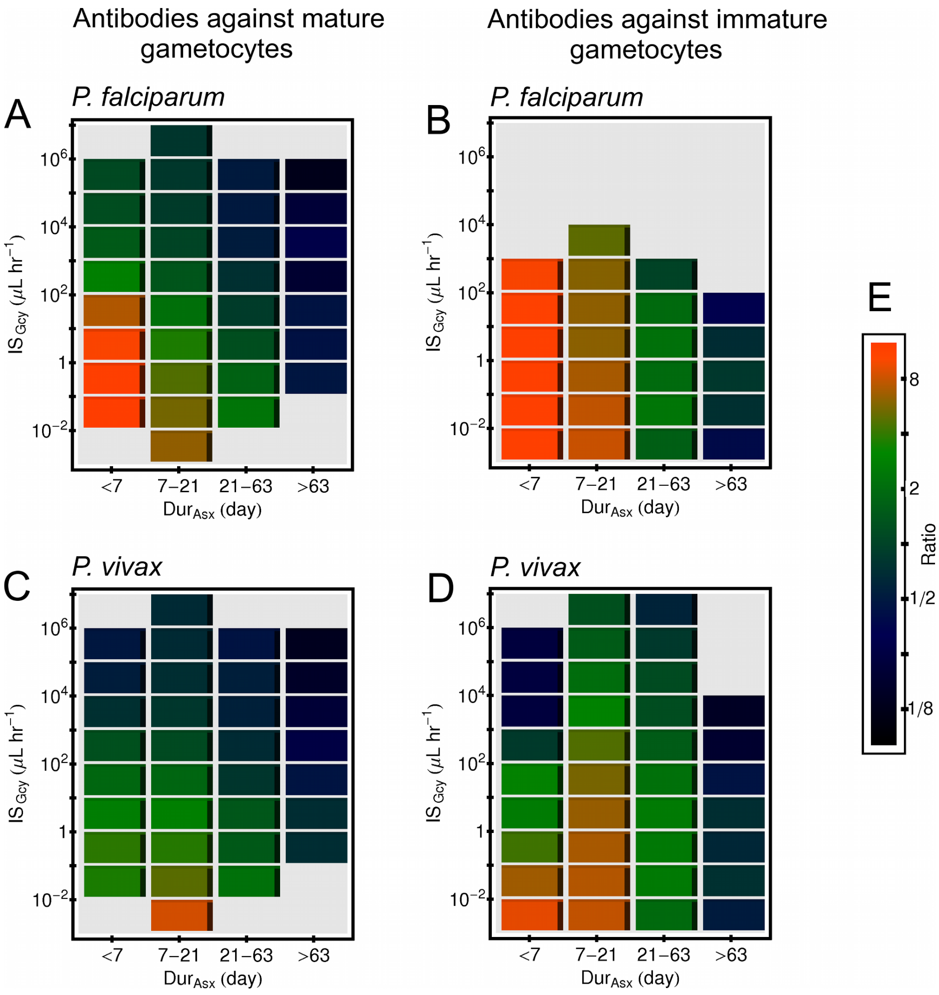

Figure S10: Effects of gametocyte-specific antibodies in simulated infection on gametocyte duration, Non-CS Models Results are from simulated infections in which the host cleared all parasites within three years from primary release of merozoites, and which also made mature gametocytes. The ratio of the duration of mature gametocytes, Dur_{MG} to the duration of the asexual forms, Dur_{Asx} was binned by Dur_{Asx}, and also by a measure of the ability of the antibody response to clear their target, IS_{Gcy}. The horizontal length of an individual block shows the bin size in Dur_{Asx}, and the vertical extent of a block show the bin size in IS_{Gcy}. Results are only shown for bins that contained 10 or more simulations. (A) Dur_{MG}/Dur_{Asx} for simulated *P. falciparum* infections in which antibodies attacked mature gametocytes and not immature forms. (B) Dur_{MG}/Dur_{Asx} for simulated *P. falciparum* infections in which antibodies attacked immature gametocytes and not mature forms. (C) Same as (A), except for simulated *P. vivax* infections. (D) Same as (B), except for simulated *P. vivax* infections. (E) Color code used for Dur_{MG}/Dur_{Asx}.

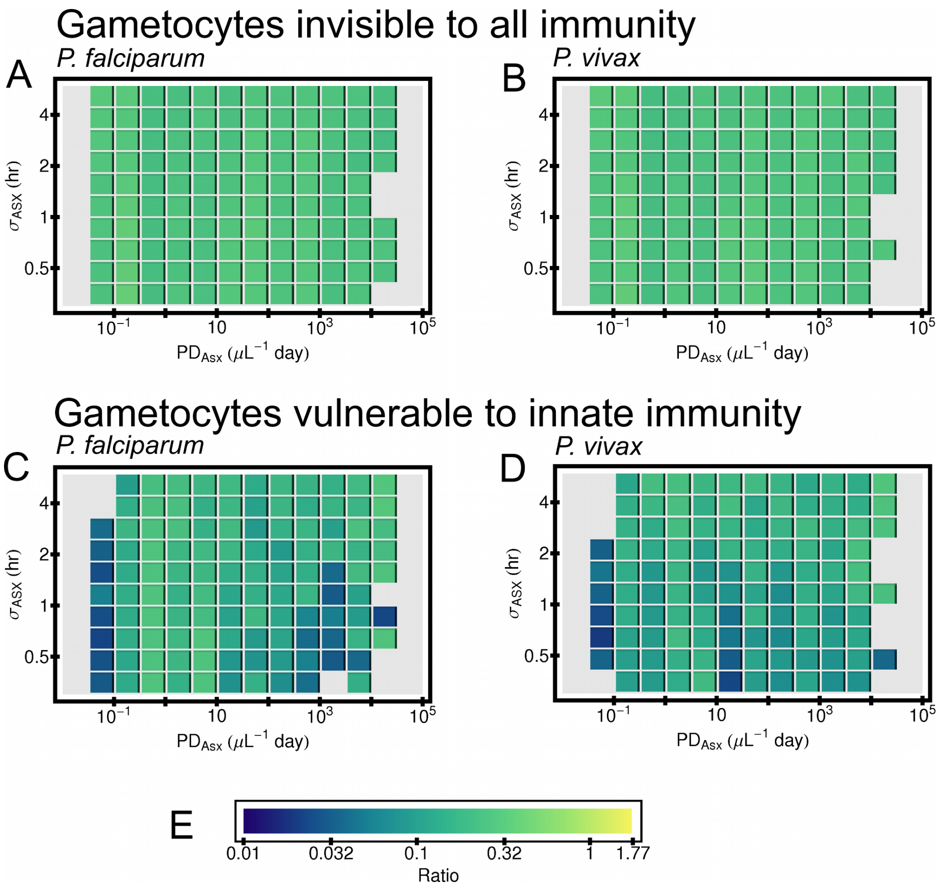

Figure S11: , Non-CS Models Results are from simulated infections in which the host cleared all parasites within three years from primary release of merozoites, and which also made mature gametocytes. Gametocytes are assumed to be invisible to acquired immunity. The ratio of the parasite days of mature gamcocytes to parasite days of asexual forms, P_{MG}/PD_{Asx}, was binned by PD_{Asx} and by the standard deviation of development time of intracellular asexual forms, σ_{Asx}. The horizontal length of an individual block shows the bin size in PD_{Asx}, and the vertical extent of a block show the bin size in σ_{Asx}. Results are only shown for bins that contained 10 or more simulations. (A) PD_{MG}/PD_{Asx} for simulated *P. falciparum* infections in which gametocytes are unaffected by host immune responses. (B) Same as (A), except for simulated *P. vivax* infections. (C) PD_{MG}/PD_{Asx} for simulated *P. falciparum* infections in which model innate response cleared mature and immature gametocytes at the same rate that it clears asexual forms. (D) Same as (C), except for simulated *P. vivax* infections. (E) Color code used for PD_{MG}/PD_{Asx}.
