# Supplementary material for: Host immune constraints on malaria transmission: insights from population biology of within-host parasites
Source: Malar J. 2013 Jun 15;12:206. doi: 10.1186/1475-2875-12-206 (PMC3691866; doi:10.1186/1475-2875-12-206)
Supplement: Additional file 2 — Second supplemental file to the report “Potential host immune constraints upon malaria transmission: insights from population biology of the within-host parasites”. This PDF file contains three discussions: (1) a brief introduction to matrix and vector notation for those unfamiliar with such notation (which helps to state the model formalism concisely), (2) the full set of equations for the model formalism, and (3) more justification as to why antibodies to immature gametocytes would be more efficient in suppressing the density of transmissible gametocytes than antibodies directly against the transmissible forms. [file 1475-2875-12-206-S2.pdf]

Additional File 2 supplemental file to the report "Potential host immune constraints upon malaria transmission: insights from population biology of the within-host parasites"

Philip G. McQueen[1,*], Kim C. Williamson[2,3] F. Ellis McKenzie[4],
1 Mathematical and Statistical Computing Laboratory, Division of Computational Bioscience, Center for Information Technology, National Institutes of Health, Bethesda, Maryland, USA
2 Loyola University of Chicago, Department of Biology, Chicago, Illinois USA
3 Laboratory of Malaria and Vector Research, National Institute of Allergy and Infectious Diseases, National Institutes of Health, Bethesda, Maryland, USA
4 Fogarty International Center, National Institutes of Health, Bethesda, Maryland, USA
∗ E-mail: mcqueenp@mail.nih.gov

Abstract

This file contains three sections of discussions to supplement the main text. The first section has a brief introduction to matrix and vector notation for those unfamiliar to such notation. (Use of this notation allows a concise representation of the system of differential equations used by this report.) The second section gives the full set of differential equation system for the model, and gives the values or the range of values of the parameters used. The third section argues why it is plausible that host antibodies to the immature gametocytes would reduce the overall mature gametocyte-days to a greater degree that antibodies directly against the mature gametocytes.

A Note on Vector and Matrix Notation

Vector and matrix notation came from linear algebra, which is concerned with solving linear systems of equations:

$$\sum_{j=1}^{N} A_{i,j} x_j \quad = \quad b_i, \quad \text{where} \quad 1 < i \leq N, \quad \text{and} \quad 1 < j \leq N. \tag{1}$$

Here the A and b factors are known quantities, and the x are unknowns to be determined. This report is not directly concerned about such systems of equations, and in fact, the systems of ordinary differential equations used for this report are highly *non-linear* in the population variables since the populations of parasite and host cells interact with each other. (For those interested in solving systems of equation such as 1, there are many outstanding textbooks on linear algebra. The book by Press, Teukolsky, *et al.* referenced in the main text gives details on numerical algorithms which solve linear algebraic systems as well as additional references.) Nonetheless, vector and matrix notation allows the theoretical population biology equations of dynamics to be represented in a compact, concise manner.

An $N \times M$ matrix A is defined as a rectangular array of numbers with N rows and M columns. The component at the intersection of row i and column j is labeled as $A_{i,j}$. Matrix addition and multiplication are defined as follows: if two matrices A and B are $N \times M$, then $\mathsf{C} = \mathsf{A} + \mathsf{B}$ is an $N \times M$ matrix such that

$$C_{i,j} \quad = \quad A_{i,j} + B_{i,j}, \quad 1 < i \leq N, \quad \text{and} \quad 1 < j \leq M. \tag{2}$$

If matrix A is $N \times P$ and matrix B is $P \times M$, then $\mathsf{C} = \mathsf{AB}$ is an $N \times M$ matrix such that

$$C_{i,j} = \sum_{k=1}^{P} A_{i,k} \times B_{k,j}, \quad 1 < i \leq N, \quad \text{and} \quad 1 < j \leq M. \tag{3}$$

A special class of matrices are $N \times 1$ arrays, called vectors in this report. Usually, the components of a vector are labelled just by the corresponding row, and the column number is dropped, since there is only one column. An $N \times 1$ vector is said to have length N. The system of equations 1 above can be written in a compact vector-matrix notation:

$$\mathsf{A}\,\boldsymbol{x} \;=\; \boldsymbol{b} \tag{4}$$

Here, the factors $A_{i,j}$ are the components of $N \times N$ matrix A, the factors x_i are the components of vector $\boldsymbol{x}$ of length N, and the b_i are the components of vector $\boldsymbol{b}$ of length N.

The system of differential equations used to model the population dynamics in this report

Equation 1 in the main text can be represented in vector-matrix format. One can think of the P_n as the components of a vector $\boldsymbol{P}$ which has length N; in a sense, this vector contains the information about the population. In addition to $\boldsymbol{P}$, define another vector $\boldsymbol{\delta}(n)$, also of length N, which has all zero components except that the nth component is 1. Define NXN matrix D which has all zero components, except that $\mathsf{D}_{i,i} = -\mathsf{D}_{i,i-1} = 1$. Then equation 1 in main text becomes

$$\boldsymbol{P}' = s(t)\,\boldsymbol{\delta}(1) - \Lambda\,\mathsf{D}\,\boldsymbol{P} \tag{5}$$

When discussing interactions between populations, the following symbols are convenient: (1) $\mathsf{g}(x)$ = greatest integer in x, (2) $\Theta(x) = 1$ if $x > 0$, 0 otherwise, (3) $L(\boldsymbol{P}) = P_N$, the last component, and (4) $T(\boldsymbol{P}) = \sum_{n=1}^{N} P_n$, the total population. Note that L and T can be thought as operators that act upon vector $\boldsymbol{P}$ and return properties of the population.

System of equations for asexual parasite population dynamics

This discussion complements the subsection "Model for asexual parasite population dynamics" in the Methods section of the main text. As mentioned in the main text, five morphologically distinct populations of asexual parasite cells where considered: (1) ring stage, (2) early trophozoites, (3) late trophozoites, (4) schizonts, and (5) merozoites. Let $\boldsymbol{RS}$, $\boldsymbol{ET}$, $\boldsymbol{LT}$, and $\boldsymbol{Sc}$ be vectors of the compartments associated with populations 1-4 respectively. The total intracellular asexual density Asx is then $Asx = T(\boldsymbol{RS}) + T(\boldsymbol{ET}) + T(\boldsymbol{LT}) + T(\boldsymbol{Sc})$. Since population (5), has a duration D_μ of just 0.1 hour, just one compartment was used for it dynamics, with μ, and take $\sigma_\mu = D_\mu$. Let t be the time since start of primary release of merozoites from the liver into the blood. Then dynamics of the asexual population are described by the following set of equations:

$$\begin{aligned}
\boldsymbol{RS}' &= (1 - r\,\Theta(Asx - Asx_{Sx}))\,\zeta\,V\,\mu\,\boldsymbol{\delta}_1 - (\chi_{Inn} + \Lambda_{Asx})\,\mathsf{D}\,\boldsymbol{RS} \\
\text{where}\quad \Lambda_{Asx} &= N_{Asx} D_{Asx}^{-1}, \quad N_{Asx} = \mathsf{g}(D_{Asx}^{2}\sigma_{Asx}^{-2}) \\
\boldsymbol{ET}' &= \Lambda_{Asx}\,L(\boldsymbol{RS})\,\boldsymbol{\delta}_1 - (\chi_{Inn} + \Lambda_{Asx})\,\mathsf{D}\,\boldsymbol{ET} \\
\boldsymbol{LT}' &= \Lambda_{Asx}\,L(\boldsymbol{ET})\,\boldsymbol{\delta}_1 - (\chi_{Inn} + \Lambda_{Asx})\,\mathsf{D}\,\boldsymbol{LT} \\
\boldsymbol{Sc}' &= \Lambda_{Asx}\,L(\boldsymbol{LT})\,\boldsymbol{\delta}_1 - (\chi_{Inn} + \chi_{Sc,Ab} + \Lambda_{Asx})\,\mathsf{D}\,\boldsymbol{Sc} \\
\mu' &= \kappa\,\Theta(t_{PR} - t) + p\,\Lambda_{Asx}\,L(\boldsymbol{Sc}) - (\zeta\,V + D_\mu^{-1})\,\mu
\end{aligned} \tag{6}$$

Here V is the density of vulnerable erythrocytes (reticulocytes only for *P. vivax*, all red blood cells for *P. falciparam*), ζ is the binding affinity of merozoites to their target blood cell population, r is the proportion of new intracellular parasites which are committed to sexual development once Asx exceeds the trigger level Asx_{Sx}, (assumed to be $0.01\mu L^{-1}$), and p is the number of merozoites released per bursting schizont after asexual division is completed within the schizont. The term $\kappa\,\Theta(t_{PR}-t)$ accounts for primary release of merozoites from the liver. As explained in the main text, $\kappa = 0.002(\mu L\, hr)^{-1}$ and $t_{PR} = 1hr$ so that 10^4 merozoites are quickly released into an adult human with blood volume $5\times10^6\mu L$. The quantity χ_{Inn} is the rate that the model innate response form the host clears the four intracellular stages. For simplicity, it is assumed for this report that the main antibody response is against the schizont stage with time-dependent clearance rate $\chi_{Sch,Ab}$.

System of equations for population dynamics of sexual forms

This discussion complements the subsection "Model for population dynamics of sexual forms" in the Methods section of the main text. As mentioned in the main text, two very different models of gametocytogenesis, the "cryptic sexual" (CS) model and the "non-cryptic sexual" (non-CS) model were considered. The equations which govern the dynamics of the CS model are stated. The ring stage, early trophozoites, late trophozoites, schizonts, and merozoites each have cryptic sexual counterparts. For each simulation, it was assumed that the values corresponding to D_{Asx}, σ_{Asx}, N_{Asx}, D_μ, p, and ζ are the same as for the asexual populations. Let $\boldsymbol{cRS}$, $\boldsymbol{cET}$, $\boldsymbol{cLT}$, and $\boldsymbol{cSc}$ be vectors of the compartments associated with intracellular cryptic sexual stages that correspond to $\boldsymbol{RS}$, $\boldsymbol{ET}$, $\boldsymbol{LT}$, and $\boldsymbol{Sc}$ respectively. Let $c\mu$ be the cryptic merozoite density. Let $\boldsymbol{IG}$ be the vector of compartments associated with the immature gametocytes. As mentioned in the main text, gametocyte duration for *P. falciparum* was taken as D_{IG} = 216hr, σ_{IG} = 24hr, and for *P. vivax*, D_{IG} = 72hr, σ_{IG} = 12hr. For simplicity, the mature gametocyte population were represented with a single compartment, $\mathcal{MG}$, with exponential decay, $D_{MG} = \sigma_{MG}$ = 156hr. It was assumed that the cryptic sexual forms are subject to the same innate clearance rate χ_{Inn} and antibody clearance rate $\chi_{Sc,Ab}$. The equations that determine the dynamics for the CS model are as follows:

$$\begin{aligned}
\boldsymbol{cRS}' &= r\Theta(Asx - Asx_{Sx})\,\zeta\, V\ \mu\boldsymbol{\delta}_1 - (\chi_{Inn} + \Lambda_{Asx})\,\mathsf{D}\,\boldsymbol{cRS} \\
\boldsymbol{cET}' &= \Lambda_{Asx}\, L(\boldsymbol{cRS})\,\boldsymbol{\delta}_1 - (\chi_{Inn} + \Lambda_{Asx})\,\mathsf{D}\,\boldsymbol{cET} \\
\boldsymbol{cLT}' &= \Lambda_{Asx}\, L(\boldsymbol{cET})\,\boldsymbol{\delta}_1 - (\chi_{Inn} + \Lambda_{Asx})\,\mathsf{D}\,\boldsymbol{cLT} \\
\boldsymbol{cSc}' &= \Lambda_{Asx}\, L(\boldsymbol{cLT})\,\boldsymbol{\delta}_1 - (\chi_{Inn} + \chi_{Sc,Ab} + \Lambda_{Asx})\,\mathsf{D}\boldsymbol{Sc} \\
c\mu' &= p\,V\Lambda_{IS}\, cSc_{N_{IS}} - (\zeta\, V + D_\mu^{-1})\, c\mu \\
\boldsymbol{IG}' &= \zeta\, c\mu\, V - (\chi_{IG,Inn} + \chi_{IG,Ab} + \Lambda_{IG})\,\mathsf{D}\,\boldsymbol{IG} \\
\text{where}\quad \Lambda_{IG} &= N_{IG}\, D_{IG}^{-1}, \\
\text{and}\quad N_{IG} &= D_{IG}^2\,\sigma_{IG}^{-2} = 81,\ \text{for } \textit{P. falciparum},\ 36\,\text{for } \textit{P. vivax} \\
\mathcal{MG}' &= \Lambda_{IG}\, IG_{N_{IG}} - (\chi_{MG,Inn} + \chi_{MG,Ab} + D_{MG}^{-1})\,\mathcal{MG} \qquad (7)
\end{aligned}$$

Here $\chi_{IG,Inn}$ and $\chi_{IG,Ab}$ are the clearance rate of the innate and antibody immune responses, respectively, upon the immature gametocytes, and $\chi_{MG,Inn}$ and $\chi_{MG,Ab}$ are the corresponding rates for the mature gametocytes. As mentioned in the main text, for a subset of simulations $\chi_{IG,Inn} = \chi_{MG,Inn} = 0$, (that is, gametocytes invisible to innate immunity), while for all other simulations, $\chi_{IG,Inn} = \chi_{MG,Inn} = \chi_{Inn}$.

The equations that determine the dynamics for the Non-CS model are as follows:

$$\begin{aligned}
\boldsymbol{IG}' &= r\,\Theta(Asx - Asx_{Sx})\,\zeta\, V\,\mu - (\chi_{IG,Inn} + \chi_{IG,Ab} + \Lambda_{IG})\,\mathsf{D}\,\boldsymbol{IG} \\
\mathcal{MG}' &= \Lambda_{IG}\, L(\boldsymbol{IG}) - (\chi_{MG,Inn} + \chi_{MG,Ab} + D_{MG}^{-1})\,\mathcal{MG} \qquad (8)
\end{aligned}$$

Here, Λ_{IG} is defined as in equation 7 above in this supplement file.

System of equations for red blood cell dynamics

This discussion complements the subsection "Model for red blood cell dynamics" in the Methods section of the main text. Three populations were used to describe the red blood cells: (1) reticulocytes, the youngest of the erythrocytes, (2) mature red blood cells, and (3) senescent red blood cells ready to be removed by phagocytosis in the spleen, liver or bone marrow. The vectors of the compartments associated with populations 1-3 are $\boldsymbol{Re}$, $\boldsymbol{Ma}$,and $\boldsymbol{Se}$ respectively. Based on hematological studies referenced in the main text, the respective durations of the stages were $D_{Re} = 36hr$ (with $\sigma_{Re} = 6hr$),), $D_{Ma} = 2796hr$ (with $\sigma_{Ma} = 148hr$), and $D_{Se} = 48hr$ (with $\sigma_{Se} = 12hr$). Letting $\mathcal{E}$ be the rate of production of new reticulocytes from the bone marrow, the dynamics of the red blood cells are described by the following set of equations:

$$\begin{aligned}
\boldsymbol{Re}' &= \mathcal{E}\,\boldsymbol{\delta}_1 - (\zeta_{Re}\,\mu + \Lambda_{Re})\,\mathsf{D}\,\boldsymbol{Re} \\
\boldsymbol{Ma}' &= \Lambda_{Re} L(\boldsymbol{Re})\,\boldsymbol{\delta}_1 - (\zeta_{Ma}\,\mu + \Lambda_{Ma})\,\mathsf{D}\,\boldsymbol{Ma} \\
\boldsymbol{Se}' &= \Lambda_{Ma} L(\boldsymbol{Ma})\,\boldsymbol{\delta}_1 - (\zeta_{Se}\,\mu + \Lambda_{Se})\,\mathsf{D}\,\boldsymbol{Se} \\
\text{where}\quad \Lambda_{Re} &= N_{Re}\,D_{Re}^{-1}, \quad N_{Re} = D_{Re}^{2}\,\sigma_{Re}^{-2} = 36, \\
\Lambda_{Ma} &= N_{Ma}\,D_{Ma}^{-1}, \quad N_{Ma} = \mathsf{g}(D_{Ma}^{2}\,\sigma_{Ma}^{-2}) = 356, \\
\text{and}\quad \Lambda_{Se} &= N_{Se}\,D_{Se}^{-1}, \quad N_{Se} = \mathsf{g}(D_{Se}^{2}\,\sigma_{Se}^{-2}) = 16.
\end{aligned} \tag{9}$$

If the parasite is *P. vivax*, $\zeta_{Ma} = \zeta_{Se} = 0$, $\zeta_{Re} = \zeta$ as defined in equation 6 above in this supplemental file. For *P. falciparum*, $\zeta_{Ma} = \zeta_{Se} = \zeta_{Re} = \zeta$ as defined in equation 6 above.

The rate of erythropoiesis, $\mathcal{E}$, has its own dynamics. Let $\mathcal{E}_0$ be the rate that the erythropoietic system in a healthy human can make new erythrocytes so that the blood density is maintained at $5 \times 10^6 \mu l^{-1}$, (or $\approx 1736\,(\mu l\,hr)^{-1}$). Define the total red blood cell density $E_T = T(\boldsymbol{Re}) + T(\boldsymbol{Ma}) + T(\boldsymbol{Se})$. For conciseness, define $\Delta\mathcal{E} = \mathcal{E} - \mathcal{E}_0$. The dynamic model for the erythrocyte source is given by

$$\begin{aligned}
\Delta\mathcal{E}' &= \begin{cases} -\lambda_{ES}(\Delta\mathcal{E} + E_T' + \delta_{Dys}\,\zeta\,\mu\,V) & : \; -E_T' - \delta_{Dys}\,\zeta\,\mu\,V < \Delta\mathcal{E}_{MX} \\ \lambda_{ES}(\Delta\mathcal{E}_{MX} - \Delta\mathcal{E}) & : \; -E_T' - \delta_{Dys}\,\zeta\,\mu\,V > \Delta\mathcal{E}_{MX} \end{cases} \\
\text{where}\quad \Delta\mathcal{E}_{MX} &= 4 \times \mathcal{E}_0 \quad \text{and} \quad \lambda_{ES}^{-1} = 48hr.
\end{aligned} \tag{10}$$

(Here V is the same as in equation 6 above.) If $\delta_{Dys} = 0$, then the rate of production of reticulocytes would increase (up to $5 \times \mathcal{E}_0$) in response to blood loss ($E_T' < 0$). The term $\delta_{Dys}\,\zeta\,\mu\,V$ is a simple model to account for dyserythropoiesis due to the parasites.

System of equations for immune response dynamics

This discussion complements the subsection"Model for immune response dynamics" in the Methods section of the main text. As stated in the main text, an actuator-attacker model was used for the immune response: the actuator is triggered when the density of a target is above some threshold, and the attacker is a factor that attempts to remove the target. The model immune responses incorporated feed-back for self-amplification then for self-limiting.

In the CS model of gametocytogenesis, dynamics of the host innate immune response are set by

$$\begin{aligned}
\mathcal{A}_{Inn}' &= \mathcal{FB}_{\mathcal{A}}\,\mathcal{FB}_{\mathcal{K}}\,\big(\Theta(\mu + c\mu - \mu_{Th})\,a_{Inn}\,\mathcal{A}_{Inn} + \lambda_{\mathcal{A}_{Inn}}\,\mathcal{A}_{Inn,0}\big) - \lambda_{\mathcal{A}_{Inn}}\,\mathcal{A}_{Inn} \\
\chi_{Inn}' &= \mathcal{FB}_{\mathcal{K}}\,\lambda_{\mathcal{A}_{Inn}}\,(\mathcal{A}_{Inn} - \mathcal{A}_{Inn,0}) - \lambda_{\chi_{Inn}}\chi_{Inn} \\
\text{where}\quad \lambda_{\mathcal{A}_{Inn}}^{-1} &= 1hr, \qquad \lambda_{\chi_{Inn}}^{-1} = 2hr, \\
\mathcal{FB}_{\mathcal{A}} &= \big(1 - (\mathcal{A}_{Inn} - \mathcal{A}_{Inn,0})\,\Delta\mathcal{A}_{Inn,Mx}^{-1}\big)\,\Theta(\Delta\mathcal{A}_{Inn,MX} - \mathcal{A}_{Inn} + \mathcal{A}_{Inn,0}), \\
\text{and}\quad \mathcal{FB}_{\mathcal{K}} &= \big(1 - \chi_{Inn}\,\chi_{Inn,Mx}^{-1}\big)\,\Theta(\chi_{Inn,Mx} - \chi_{Inn}).
\end{aligned} \tag{11}$$

The self-amplification parameter a_{Inn} is taken to have value 10, and the background actuator level $\mathcal{A}_{Inn,0}$ is taken as $0.1\mu l^{-1}$. The $\mathcal{FB}$ factors enforce self-limiting feedback. As mentioned in the main text, the limit on growth of the actuator is set by parameter $\Delta\mathcal{A}_{Inn,Mx} = 10\mu l^{-1}$. The limit on growth of the attacker is set by maximum clearance rate $\chi_{Inn,Mx}$, and was also varied from simulation to simulation. The threshold density of merozoites that triggers this response is μ_{Th} which is also varied from simulation to simulation. (For the non-CS model simulations, $\Theta(\mu + c\mu - \mu_{Th})$ in equation 11 above is replaced with $\Theta(\mu - \mu_{Th})$.)

The model antibody responses incorporate an addition component, the delay stage (as shown in Figure 1 of main text). Let $\boldsymbol{G}$ be the vector consisting of the components of the delay stage, and let $\mathcal{A}_{Ab,Tar}$ and $\chi_{Ab,Tar}$ be the actuator and attacker phases, respectively, of an antibody response against a targeted stage Tar. For the delay stage, its duration was taken as $D_G = 96hr$ with $\sigma_G = 9.6hr$. The dynamics for each of the antibody responses are determined by the following system:

$$\begin{aligned}
\mathcal{A}'_{Ab,Tar} &= \mathcal{FB}_{\mathcal{A}}\,\mathcal{FB}_{\mathcal{K}}\left(\Theta(Tar - Tar_{Th})\,a_{Ab}\,\mathcal{A}_{Ab,Tar} + \lambda_{\mathcal{A}_{Ab}}\,\mathcal{A}_{Ab,0}\right) - \lambda_{\mathcal{A}_{Ab}}\,\mathcal{A}_{Ab,Tar} \\
\boldsymbol{G'} &= \lambda_{\mathcal{A}_{Ab}}\,\Theta(\mathcal{A}_{Ab,Tar} - \mathcal{A}_{Ab,0})\left(\mathcal{A}_{Ab,Tar} - \mathcal{A}_{Ab,0}\right)\boldsymbol{\delta}_1 - \Lambda_G\,\mathsf{D}\,\boldsymbol{G} \\
\chi'_{Ab,Tar} &= \mathcal{FB}_{\mathcal{K}}\,\lambda_G\,L(\boldsymbol{G}) - \lambda_{\chi_{Ab,Tar}}\chi_{Ab,Tar} \\
\text{where}\quad \lambda_G &= N_G\,D_G^{-1} \quad N_G = D_G^2\,\sigma_G^{-2} = 100, \quad \lambda^{-1}_{\mathcal{A}_{Ab}} = 1hr, \\
\mathcal{FB}_{\mathcal{A}} &= \left(1 - (\mathcal{A}_{Ab,Tar} - \mathcal{A}_{Ab,0})\,\Delta\mathcal{A}^{-1}_{Ab,MX}\right)\Theta(\Delta\mathcal{A}_{Ab,MX} - \mathcal{A}_{Ab,Tar} + \mathcal{A}_{Ab,0}), \\
\text{and}\quad \mathcal{FB}_{\mathcal{K}} &= \left(1 - \chi_{Ab,Tar}\,\chi^{-1}_{Ab,Tar,Mx}\right)\Theta(\chi_{Ab,Tar,Mx} - \chi_{Ab,Tar}). \qquad (12)
\end{aligned}$$

For all antibody responses modeled, the self-amplification parameter $a_{Ab} = 1$, the background actuator level $\mathcal{A}_{Sc,Ab,0} = 0.1\mu l^{-1}$, and $\Delta\mathcal{A}_{Ab,MX} = 10\mu l^{-1}$. Tar is the density of the target of the response, and Tar_{Th} is the threshold density that triggers the response. As mentioned in the main text, all simulations have an antibody response against schizonts (and crypto-sexual schizonts in the CS model). Some will have a response against mature gametocytes and some against immature gametocytes. The parameters μ_{Th} and $\chi_{Inn,Mx}$ in equation 11, and Tar_{Th} and $\chi_{Ab,Tar,Mx}$ from equation 12 are varied from simulation to simulation.

How Antibodies to Immature Gametocytes Affect the Density of Mature Gametocytes

In the main text it is stated, "Antibodies against immature gametoctyes tend to be more effective in reducing the density of transmissible gametocytes than antibodies directly against the transmissible forms." Although the models of host immune dynamics are complicated (and real immune reactions are even more complicated), on can understand this result by using a simple calculation: let g_0 be the number of immature gametocytes created during a burst of parasitemia. Assume that an immune response would attack this population with a constant removal rate χ_K until it reaches maturity. Then the size of this cohort decreases exponentially until maturity is reached. After maturity the population decays exponentially with time constant D_{MG}. The contribution of the cohort to PD_{MG} is

$$\begin{aligned}
\delta PD_{MG} &= g_0\,exp(-D_{IG}\chi_K)\int_0^\infty exp(-t/D_{MG})dt \\
&= g_0\,exp(-D_{IG}\chi_K)D_{MG} \\
&= g_0\,exp(-9days \times \chi_K) \times 6.5days \quad \text{for} \quad P.\ falciparum, \\
&= g_0\,exp(-3days \times \chi_K) \times 6.5days \quad \text{for} \quad P.\ vivax. \qquad (13)
\end{aligned}$$

On the other hand, if the cohort of immature gametocytes are not affected by host immune responses, but then are exposed to an immune response with a constant killing rate χ_K after reaching maturity, the contribution to PD_{MG} is

$$\begin{aligned} \delta PD_{MG} &= g_0 \int_0^\infty exp(-t \times (D_{MG}^{-1} + \chi_K)dt \\ &= g_0 \times D_{MG}\Big(1 + D_{MG} \times \chi_K\Big)^{-1} \\ &= g_0 \times 6.5days\Big(1 + 6.5days \times \chi_K\Big)^{-1} \end{aligned} \quad (14)$$

Thus the ratio r_χ of δPD_{MG} calculated assuming that only immature gametocytes are attacked by an immune response with killing rate χ_K to δPD_{MG} calculated assuming that only mature gametocytes are attacked with a killing rate of the same strength is

$$\begin{aligned} r_\chi &= exp(-9days\chi_K)\Big(1 + 6.5days \times \chi_K\Big) \quad \text{for} \quad P.\ falciparum, \\ &= exp(-3days\chi_K)\Big(1 + 6.5days \times \chi_K\Big) \quad \text{for} \quad P.\ vivax. \end{aligned} \quad (15)$$

Figure S12 shows the value of this ratio as a function of χ_K for infections with either *P. falciparum* or *P. vivax*. It can be seen that r_χ is rapidly suppressed below one as χ_K grows, especially for *P. falciparum* infection. Although the clearance rate of the immune responses of the models discussed in the main text are not constant, nonetheless one can see that the mature gametocyte population, especially that of *P. falciparum*, would be very sensitive to immune pressures on the immature gametocytes.

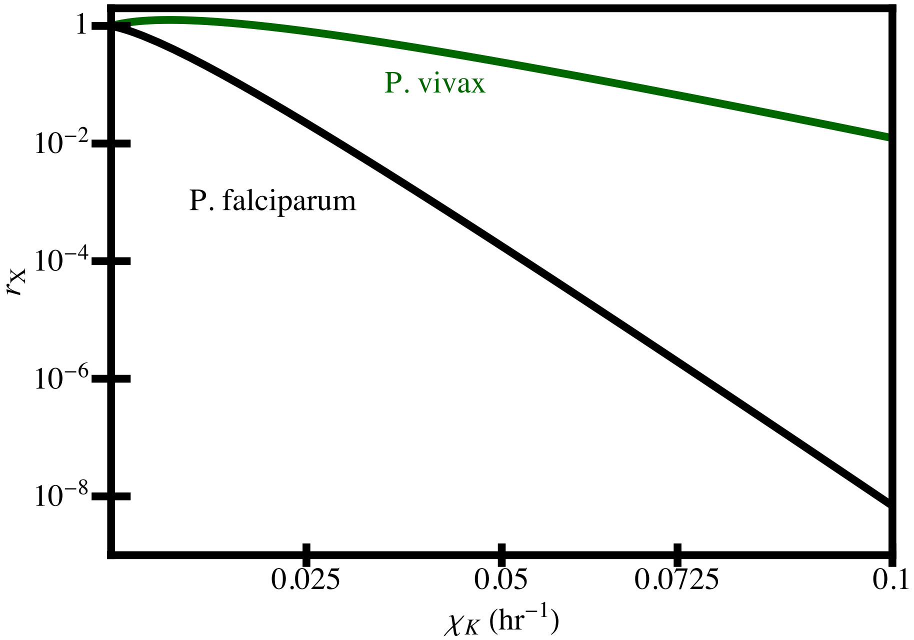

Figure S12: Plot of Ratio of Immune Suppression r_χ versus Immune Clearance Rate χ_K
Note: a clearance rate of $0.05hr^{-1}$ corresponds to $1.2day^{-1}$.

Tables

Table S1 - Parameters varied from simulation to simulation

Abbreviations: Sc: schizont, IG: immature gametocyte, MG: mature gametocyte, IG Ab+(-) antibodies to immature gametocytes present (absent), MG Ab+(-): antibodies to mature gametocytes present (absent).

Parameter	Equation where used	Range in values	Model Class
$\log(\sigma_{Asx} \times hr^{-1})$	6	$\log(0.3) - \log(5.9)$	All
δ_{Dys}	10	$0 - 10$	All
$\log(\mu_{Th} \times \mu L)$	11	$\log(10^{-5}) - \log(10)$	All
$\log(\chi_{Inn,Mx} \times hr)$	11	$\log(0.05) - \log(50)$	All
$\log(Sc_{Th} \times \mu L)$	12	$\log(10^{-5}) - \log(10)$	All
$\log(\chi_{Ab,Sc,Mx} \times hr)$	12	$\log(0.05) - \log(50)$	All
$\lambda_{\chi_{Ab,Sc}}$	12	$168hr - 8760hr$	All
$\log(IG_{Th} \times \mu L)$	12	$\log(10^{-5}) - \log(10)$	IG Ab+, MG Ab-
$\log(\chi_{Ab,IG,Mx} \times hr)$	12	$\log(0.05) - \log(50)$	IG Ab+, MG Ab-
$\lambda_{\chi_{Ab,IG}}$	12	$168hr - 8760hr$	IG Ab+, MG Ab-
$\log(MG_{Th} \times \mu L)$	12	$\log(10^{-5}) - \log(10)$	IG Ab-, MG Ab+
$\log(\chi_{Ab,MG,Mx} \times hr)$	12	$\log(0.05) - \log(50)$	IG Ab-, MG Ab+
$\lambda_{\chi_{Ab,MG}}$	12	$168hr - 8760hr$	IG Ab-, MG Ab+
